# Mechanisms of Egg Yolk Formation and Implications on Early Life History of White Perch (*Morone americana*)

**DOI:** 10.1371/journal.pone.0143225

**Published:** 2015-11-18

**Authors:** Justin Schilling, Philip L. Loziuk, David C. Muddiman, Harry V. Daniels, Benjamin J. Reading

**Affiliations:** 1 Department of Applied Ecology, North Carolina State University, Raleigh, North Carolina, United States of America; 2 W. M. Keck FTMS Laboratory for Human Health Research, Department of Chemistry, North Carolina State University, Raleigh, North Carolina, United States of America; 3 Center for Comparative Medicine and Translational Research, North Carolina State University, Raleigh, North Carolina, United States of America; Glasgow Caledonian University, UNITED KINGDOM

## Abstract

The three white perch (*Morone americana*) vitellogenins (VtgAa, VtgAb, VtgC) were quantified accurately and precisely in the liver, plasma, and ovary during pre-, early-, mid-, and post-vitellogenic oocyte growth using protein cleavage-isotope dilution mass spectrometry (PC-IDMS). Western blotting generally mirrored the PC-IDMS results. By PC-IDMS, VtgC was quantifiable in pre-vitellogenic ovary tissues and VtgAb was quantifiable in pre-vitellogenic liver tissues however, neither protein was detected by western blotting in these respective tissues at this time point. Immunohistochemistry indicated that VtgC was present within pre-vitellogenic oocytes and localized to lipid droplets within vitellogenic oocytes. Affinity purification coupled to tandem mass spectrometry using highly purified VtgC as a bait protein revealed a single specific interacting protein (Y-box binding protein 2a-like [Ybx2a-like]) that eluted with suramin buffer and confirmed that VtgC does not bind the ovary vitellogenin receptors (LR8 and Lrp13). Western blotting for LR8 and Lrp13 showed that both receptors were expressed during vitellogenesis with LR8 and Lrp13 expression highest in early- and mid-vitellogenesis, respectively. The VtgAa within the ovary peaked during post-vitellogenesis, while VtgAb peaked during early-vitellogenesis in both white perch and the closely related striped bass (*M*. *saxatilis*). The VtgC was steadily accumulated by oocytes beginning during pre-vitellogenesis and continued until post-vitellogenesis and its composition varies widely between striped bass and white perch. In striped bass, the VtgC accounted for 26% of the vitellogenin-derived egg yolk, however in the white perch it comprised only 4%. Striped bass larvae have an extended developmental window and these larvae have yolk stores that may enable them to survive in the absence of food for twice as long as white perch after hatch. Thus, the VtgC may play an integral role in providing nutrients to late stage fish larvae prior to the onset of exogenous feeding and its composition in the egg yolk may relate to different early life histories among this diverse group of animals.

## Introduction

Vitellogenins (Vtgs) are the predominant egg yolk precursors in oviparous vertebrates. Vtgs are synthesized in the liver under the influence of estrogen, secreted into the bloodstream as ~500 kDa homodimers, and bind specific receptors on the surface of growing oocytes during a process termed *vitellogenesis*. Multiple vitellogenins have been described in a number of fish species including mummichog (*Fundulus heteroclitus*) [1 and 2], barfin flounder (*Verasper moseri*) [3], haddock (*Melanogrammus aeglefimus*) [4], zebrafish (*Danio rerio*) [5 and 6], mosquitofish (*Gambusia affinis*) [7 and 8], red seabream (*Pagrus major*) [9], Atlantic halibut (*Hippoglossus hippoglossus*) [10 and 11], gray mullet (*Mugil cephalus*) [12 and 13], goldsinny wrasse (*Ctenolabrus rupestris*) [14], striped bass (*Morone saxatilis*) [15], and white perch (*Morone americana*) [16 and 17]. In addition to their roles in providing the nutrients for developing embryos, Vtgs also have been shown to be immune-relevant molecules involved in hemagglutinating actions and in host defense against microbes including bacteria and viruses [18–22].

Complete type Vtgs of Acanthomorph fish species are classified as either VtgAa or VtgAb based upon their structures using the nomenclature system proposed by Finn and Kristofferson [11]. Complete type Vtgs contain five distinct yolk protein domains: Lipovitellin heavy chain (LvH), phosvitin (Pv), lipovitellin light chain (LvL), beta-component (β'-c), and C-terminal domain (C-t). The third form of Acanthomorph vitellogenin, VtgC, is an incomplete type that lacks or has reduced Pv, β'-c, and C-t domains [5 and 11]. In marine fishes that spawn floating eggs, for instance, the yolk protein domains of these three Vtgs undergo varying degrees of proteolysis into free amino acids (FAAs) during ovarian maturation. The LvH domain of VtgAa (LvHAa), for instance, is extensively proteolyzed, while those of VtgAb (LvHAb) and VtgC (LvHC) remain largely intact [3, 4, and 11]. The FAAs derived from VtgAa form an osmotic gradient that contributes to oocyte hydration and egg buoyancy in fish species that spawn pelagic eggs [3]. These LvHAa-derived FAAs also serve as diffusible nutrients during embryogenesis while LvHAb remains largely intact and is consumed by later stage embryos [3, 8, 9, 12, 13]. The LvHC is the last remaining Vtg-derived yolk component to be consumed by developing yolk sac larvae [7]. Varying ratios of these three Vtg forms accumulated in the egg yolk in turn lead to varying degrees of oocyte hydration and egg buoyancy that may relate to spawning strategies and likely have influence for early life history and development [17, 23, 24].

Complete type Vtgs (VtgAa and VtgAb) have been shown to bind two lipoprotein receptors on the surface of growing oocytes, LR8 and Lrp13. We have recently shown that in white perch VtgAa binds primarily to Lrp13 while VtgAb binds to LR8 [25–27]. The exact mode of oocyte entry for VtgC has remained unclear, however it has not been shown to bind either of these oocyte membrane lipoprotein receptors [26].

Previous studies have provided both semiquantitative and quantitative analyses of vitellogenins in assorted tissues of oviparous vertebrates including fishes, sea turtle, and chickens by mass spectrometry (MS)-based proteomics [15, 16, 28–33]. This study represents, to the best of our knowledge, the first absolute quantification of vitellogenins by PC-IDMS in liver, plasma, and ovary tissues across an entire reproductive cycle of any egg-laying animal. In addition, VtgC localization within the growing oocyte is illustrated by immunohistochemistry and an apparent specific VtgC-interacting protein is identified by affinity purification coupled to tandem mass spectrometry. To date, no study has demonstrated interaction of VtgC with ovary proteins or provided a possible explanation for its entry into the oocyte aside from the hypothesis of fluid phase endocytosis [26, 27]. Expression levels of the three white perch vitellogenins and the two vitellogenin receptors across the annual reproductive cycle are also shown by western blotting. Furthermore, we explore possible adaptive consequences of the quantitative Vtg ratios of the white perch in comparison to those of the closely related striped bass.

## Materials and Methods

### Tissue Collection

Adult female white perch were reared under natural photothermal conditions at the NC State University Pamlico Aquaculture Field Laboratory (Aurora, NC) [34]. All experiments in the present study were approved by the North Carolina State University Institutional Animal Care and Use Committee (IACUC) protocol 13-041-A. Liver, plasma, and ovary were sampled at four time points across one year: August, November, February, and May. At each time point, three fish (N = 3 biological replicates) were anesthetized with Finquel MS-222 (Argent Chemical Laboratories, Redmond, WA) and tissues individually collected and separately stored. Blood was collected from the caudal vein by heparinized needle and syringe and transferred into 12 x 75 mm tubes containing 25 μL of aprotinin solution (40 TIU/mL aprotinin in 0.9% NaCl) (Sigma Aldrich, St. Louis, MO). Following centrifugation (5 min at 2,000 × g), the plasma was stored at -80°C until use. Liver and ovary tissues were collected and stored at -80°C until use. Additional ovary tissue was fixed in Bouin’s solution in preparation for histology and immunohistochemistry (IHC). The white perch is a multiple-clutch, group-synchronous spawner and therefore the ovaries were staged based upon the most advanced clutch of oocytes at each of the four sampling time points as previously described [34]. As such, the white perch ovary contains a distribution of all of the different stages of oocytes from primary growth up to those representing the most advanced (top clutch) that will be ovulated during the upcoming spawning cycle.

### Filter-Aided Sample Preparation of White Perch Tissues

Plasma and Dounce-homogenized liver and ovary samples were thawed and diluted with Tris-buffered saline (20 mM Tris-HCL pH 8.0, 150 mM NaCl, and 2 mM CaCl_2_) to a final protein concentration of 1 mg/mL as determined by Bradford assay [35, 36]. Liver, plasma, and ovary samples from each fish at each time point were individually prepared for tandem mass spectrometry using a modified filter-aided sample preparation (FASP) protocol [16]. Samples were incubated for 30 min at 56°C with 15 μL of 50 mM DTT per 200 μL (200 μg) of sample to reduce disulfide bonds. Proteins were denatured by adding 200 μL of 8 M urea in 0.1 M Tris-HCl pH 8.0 to each sample. Each sample was then transferred onto a Vivacon 500 30 kDa MW cutoff filter (Sartorius Stedim Biotech, Goettingen, Germany) and centrifuged at 14,000 × g for 15 min at 21°C. To ensure denaturation, an additional 200 μL of 8 M urea in 0.1 M Tris-HCl pH 8.0 was added to each sample before centrifuging again as above. Each sample was on-filter alkylated by adding 64 μL of 200 mM iodoacetamide (50 mM final) prepared in 8 M urea. Samples were incubated in the dark at 37°C for 1 hr and then centrifuged at 14,000 × g for 15 min at 21°C. Each filter was washed three times with 100 μL of 2 M urea/10 mM CaCl_2_ by centrifugation for 10 min at 14,000 × g followed by three washes with 100 μL of 0.1 M Tris pH 7.5. Each sample was then placed in a new centrifuge tube and modified trypsin freshly prepared in 0.1 M Tris pH 7.5 was added to each sample at an enzyme-to-protein ratio of 1: 5. Following eight hours of digestion at 37°C, trypsinization was quenched with 50 μL of 0.001% zwittergent 3–16 (Calbiochem, La Jolla, CA)/1% formic acid and tryptic peptides were collected by centrifugation at 14,000 × g for 10 min at 21°C. A second quench/wash step was carried out to maximize tryptic peptide recovery using 400 μL of 0.001% zwittergent/1% formic acid and centrifugation at 14,000 × g for 30 min at 21°C. Samples were dried using a SpeedVac (Thermo Fisher Scientific, San Jose, CA) and stored at -20°C.

### LC-MS/MS Materials

Unless otherwise stated, all reagents were purchased from Sigma-Aldrich (St. Louis, MO). All solvents were HPLC-grade from Honeywell Burdick & Jackson (Muskegon, MI).

### Stable Isotope-Labeled Peptide Standards and Transition Characterization

Stable isotope-labeled (SIL) surrogate peptides uniquely identifying their respective protein were synthesized by New England Peptide (Gardner, MA) (S1 Table). Newly obtained standards from New England Peptide were subjected to direct infusion into a TSQ Quantum triple quadrupole mass spectrometer (Thermo Fisher Scientific, San Jose, CA) at 50 μM in order to identify the most abundant charge state for each peptide precursor. The most abundant precursor of each standard was fragmented and the 6 most abundant product ions of each precursor selected for further characterization. Collision energy optimization experiments were performed by optimization in the tune software, testing a range of collision energies and choosing the energy for which maximum signal intensity of each peptide was obtained.

### LC-MS/MS Analysis

All biological replicates were analyzed in triplicate (technical replicates) using an Eksigent nanoLC-2D (AB SCIEX, Framingham, MA) equipped with an AS1 autosampler and coupled with TSQ Quantum triple quadrupole mass spectrometer (Thermo Fisher Scientific, San Jose, CA) via a direct inject configuration in which 10 μL was injected onto a C18 reverse phase analytical column, 150 X 0.5 mm (Agilent Technologies, Santa Clara, CA) both packed with Zorbax SB-C18 (5 μm particle size). Mobile phases A and B were composed of water/acetonitrile/formic acid (98/2/0.2% and 2/98/0.2%, respectively). The sample was loaded at 12 μL/min for 3 min using mobile phase A. A 30 min elution was then performed at a flow rate of 16 μL/min utilizing a 5–40% mobile phase B gradient. Compounds were ionized using an ion max source with a spray potential of 3.5 kV, a sheath gas pressure of 30 units, a capillary temperature of 270°C, and a skimmer offset of 8 V. Targets were analyzed using selected reaction monitoring mode. Instrument parameters included an argon collision gas pressure of 1.5 mTorr, a full width half maximum of 0.7 *m/z* for Q1 and Q3, a scan width of 0.01 *m/z*, a scan time of 0.050 sec/transition and a chrom filter peak width of 45 seconds. Data from targeted LC-MS/MS experiments were imported into Skyline v.2.5.0.6079 [37] where reproducible co-elution of native and SIL peptides along with their respective transitions was used to confirm the presence of the target peptide. Transitions that did not co-elute or appeared to not follow the conserved fragmentation pattern of the internal standard were flagged as contaminated and excluded from further analysis. Peak integration was performed after manual adjustment of integration windows in order to ensure consistent integration across injections. Data were then exported into Excel. Peak area ratios of native-to-SIL peptides were multiplied by the amount of each SIL peptide added during digestion to obtain absolute quantities in femtomoles (fmol) of target protein/μg of total protein (S1 File). Since the amount of VtgC detected in the pre-vitellogenic ovary samples was toward the lower limit of confident quantification, these samples were assayed twice (S1 File).

### Experimental Replication and Statistical Analysis

PC-IDMS data from three technical replicates of each independent biological replicate were averaged and these values for the three biological replicates at each time point were analyzed by ANOVA using JMP Pro 10 (SAS Institute, Inc., Cary, NC) and employed Tukey’s Honestly Significant Difference (HSD) post hoc correction. Mean values were considered statistically different when P ≤ 0.05.

### Antibody Production

Antibodies against the two known white perch vitellogenin receptors (LR8 and Lrp13) [23] and the three vitellogenins (VtgAa, VtgAb, and VtgC) were raised against two synthetic peptides for each protein in chickens (GeneTel, Madison, WI). Great care was taken to select highly unique antigenic sequences for each target protein (S2 Table). For the white perch Vtgs, each antigenic peptide was selected from the LvH domain, as our previous studies have shown that this region undergoes less proteolysis than the other domains in this species [23, 32]. The resulting white perch vitellogenin antibodies are referred to hereafter as α-White Perch VtgAa, α-White Perch VtgAb, and α-White Perch VtgC. Preimmune chicken IgY from each immunized hen was also purified to serve as a negative control for western blotting and IHC validation.

### Western Blotting

While each tissue from each fish was individually prepared and analyzed by LC-MS/MS, samples from the same tissues and time points were pooled for western blotting. Samples were resuspended and boiled in Laemmli’s buffer and electrophoresed under reducing and denaturing conditions through BIO-RAD Mini-PROTEAN TGX 4–15% Precast Gels with 10-well comb and 30 μL per well capacity (Catalog # 456–1083). All samples were loaded at 15 μg total protein per well. Molecular weights were calculated based upon the migration distances of the BIO-RAD Kaleidoscope Precision Plus Protein Standards (Catalog # 161–0375). Primary antibodies were used at 1: 5,000 and R&D Systems biotinylated goat α-chicken IgY secondary antibody (Catalog # BAF010) was used at 1: 10,000. Blots were developed using Vector Laboratories VECTASTAIN Elite ABC-peroxidase per kit (Catalog # PK-6100) instructions.

### White Perch Vitellogenin C Purification

White perch vitellogenin C (VtgC) was purified from 1.8 mL of 17β-estradiol (E_2_)-induced male white perch plasma [38] using a combination of anion exchange chromatography and size exclusion chromatography [26]. Briefly, pooled E_2_-induced plasma was diluted in an equal volume of 20 mM Tris-Bis-Propane, pH 9.0 containing 150 mM NaCl, 3.5 TIU/L aprotinin, 5% sucrose and applied to a POROS50 column. While complete type vitellogenins (VtgAa and VtgAb) bound to the POROS50 resin, VtgC did not and was present in the flow through [26]. The flow through material was concentrated using a Centricon 10 kDa MW cutoff filter and applied to Superdex 200 gel filtration column in order to separate VtgC from remaining contaminating proteins. VtgC purity was assessed by Coomassie total protein staining and α-White Perch VtgC western blotting.

### White Perch Vitellogenin C Affinity Purification Coupled to Tandem Mass Spectrometry

Fractions containing highly purified white perch VtgC were used for an affinity purification coupled to tandem mass spectrometry experiment (AP-MS/MS). Approximately 1 mg of highly purified white perch VtgC in 3 mL of binding buffer (20 mM Tris-HCl, 2 mM CaCl_2_, and 150 mM NaCl, pH 8.0, containing 1 mM phenylmethyl-sulfonyl fluoride and 4 IU/L aprotinin) was coupled to 1 mL of Affi-Gel 15 slurry (~500 μl resin) per kit instructions. An uncoupled control resin was generated by incubating 1 mL of Affi-Gel 15 slurry (~500 μl resin) with 3 mL of binding buffer. Following coupling, any remaining reactive esters were blocked by adding 5 μl of 1 M ethanolamine HCl (pH 8.0) to each resin bed on a rotating wheel at 21°C for 1 hr. Both resin beds were then equilibrated with 10 column volumes of binding buffer. Solubilized membrane proteins were prepared from 5 g of vitellogenic white perch ovaries according to our previous studies [26 and 27] and incubated individually with control and VtgC-labeled affinity media for 4 hrs at 4°C in batch on a rotating wheel. The VtgC-labeled and unlabeled resins incubated with ovary solubilized membrane fraction were then washed with 15 volumes of binding buffer at 4°C prior to elution with 20 mM Tris-HCl, 10 mM suramin, 5 mM EDTA, 150 mM NaCl, pH 6.0. The column was equilibrated for 30 min at 4°C before eluting proteins with 5 volumes of elution buffer (~1 mL). After additional washing with 15 volumes of binding buffer at 4°C, a second elution step followed using 20 mM Tris-HCl, 5 mM EDTA, 1.5 M NaCl, pH 6.0 with which the resin was equilibrated for 30 min at 4°C before eluting proteins with 5 volumes of elution buffer (~1 mL).

### White Perch Vitellogenin C AP-MS/MS Filter-Aided Sample Preparation

Elution fractions were reduced with DTT at a final concentration of 5mM at 37°C for 30 mins and then mixed with 200 μl of 8 M urea in 0.1 M Tris-HCL pH 7.0 and placed atop Vivacon 500 30 kDa MW cutoff filters and centrifuged at 14,000 x g for 15 mins. An additional 200 μl of 8 M urea in 0.1 M Tris-HCL pH 7.0 was added to each filter unit and they were centrifuged again at 14,000 x g for 15 mins. The flow through was discarded from each collection tube. Samples were alkylated with 100 μl of 0.05 M iodoacetamide prepared in 8 M urea added to each sample, mixed for 1 min and incubated in the dark at 21°C for 20 mins before centrifuging at 14,000 x g for 15 mins. Samples were further denatured with 100 μl 8 M urea by centrifuging at 14,000 x g for 10 mins. This was repeated two additional times. Samples were then washed with 100 μl of 0.1 M Tris-HCL and centrifuged at 14,000 x g for 15 mins. This was repeated twice. A fresh collection vial was placed beneath each filter unit and 40 μl 0.1 M Tris-HCl containing modified trypsin (1: 100 ratio of trypsin: total protein) was added to each sample and mixed for 1 min. Filter units were then sealed with parafilm and incubated at 37°C for 18 hrs. Following trypsinization, 1 μl of 2% formic acid was added to each sample. All samples were then centrifuged at 14,000 x g for 15 mins. To maximize recovery of tryptic peptides, an additional 40 μl of 0.1 M Tris-HCl was added atop each filter unit and they were again centrifuged at 14,000 x g for 15 mins. Final trypsinized protein concentrations were measured using a Nanodrop at A280 (Thermo Scientific, Wilmington, DE).

### White Perch Vitellogenin C AP-MS/MS NanoReversed Phase LC-MS/MS

Protein concentrations of the digests were obtained using a Nanodrop at A280 (Thermo Scientific, Wilmington, DE). Samples were reconstituted to a protein concentration of 0.2 μg/μL using mobile phase A (98/2/0.2% water/acetonitrile/ formic acid), and a total of 5 μL was injected. Separation of peptides was performed using a Thermo Scientific EASY nLC II (Thermo Scientific, San Jose, CA) in line with a cHiPLC nanoflex system (AB Sciex, Framingham, MA), coupled to an LTQ-Orbitrap XL mass spectrometer (Thermo Scientific, San Jose, CA) [28]. A vented column configuration, a ChromXP C18-CL 3 μm trap column, and a ChromXP C18-CL 75 μm × 15 cm analytical column were used for these experiments. A flow rate of 350 nL/min and initial condition of 2% mobile phase B (2/98/0.2% water/acetonitrile/formic acid) was increased to 35% over 201 min, then steeply ramped to 95% mobile phase B over 10 min and maintained at 95% mobile phase B for 10 min to wash the column. The column was then equilibrated at 2% mobile phase B for 12 min. Each sample was analyzed in triplicate. Raw files were converted to “.mgf” using Proteome Discoverer (Thermo Scientific, San Jose, CA). Resulting “.mgf” files were searched using Sequest HT against a target database containing the striped bass ovary transcriptome (GenBank: SRX007394) translated in all six open reading frames and combined into one FASTA file that also contained the protein sequences for the white perch VtgAa, VtgAb, and VtgC (GenBank Accession DQ020120.1, DQ020121.1, and DQ020122.1, respectively). Sequences corresponding to human keratins and porcine trypsin were also included in the database. The data were searched using the following parameters: trypsin as the enzyme which performs *in silico* digestion of the target database proteins at arginine (R) and lysine (K) residues, fixed carbamidomethyl modification of cysteine residues, variable oxidation of methionine, variable deamidation of asparagine and glutamine, maximum of 2 missed cleavages, ppm precursor tolerance, and 0.06 Da MS/MS tolerance. Data were filtered at a 1% peptide FDR using percolator. Precursor ion area detector node was used to perform label-free quantification by peak area.

## Results

### Histology and Oocyte Staging

**Fig 1:**
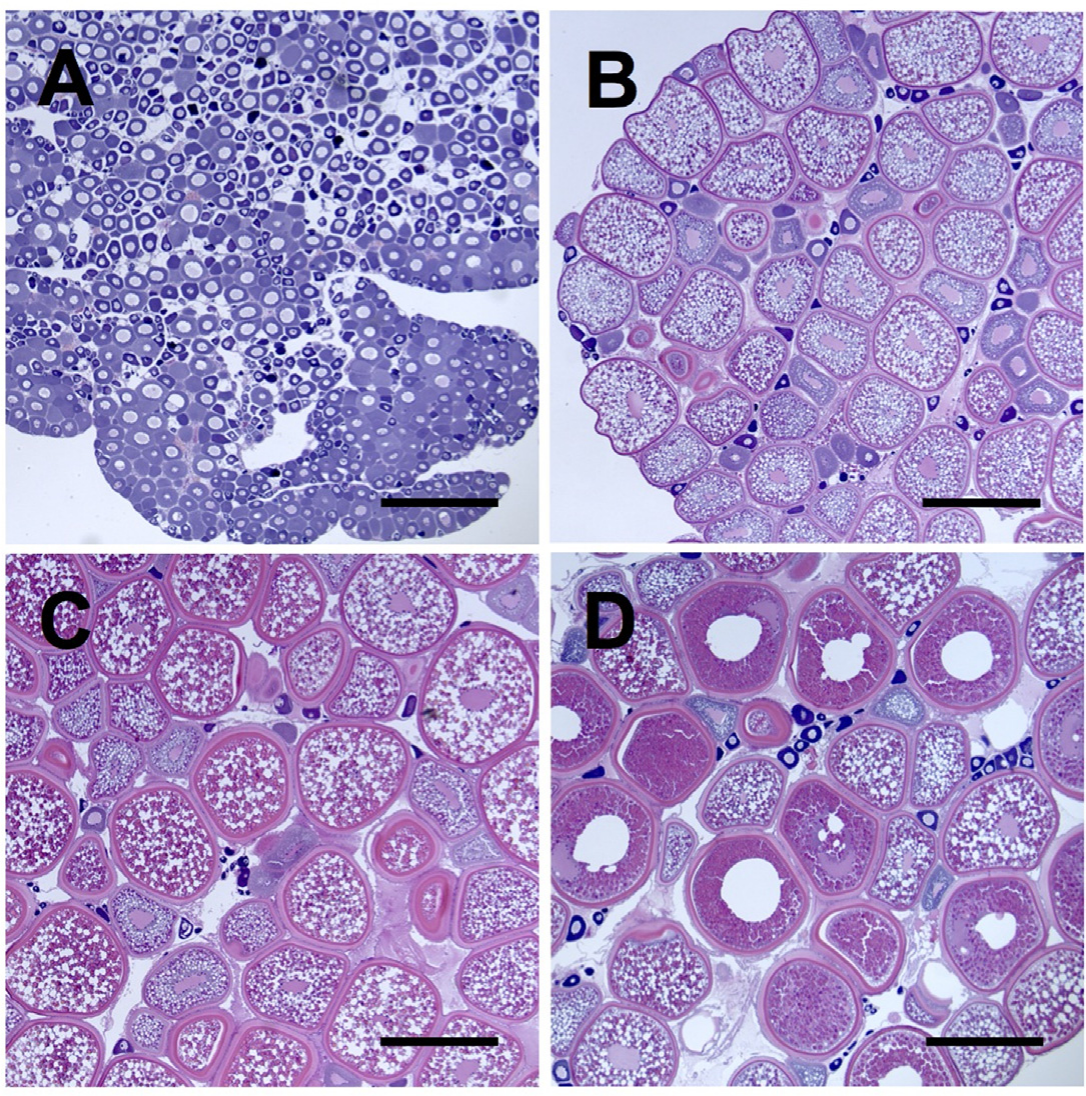
Hematoxylin and eosin staining of representative white perch ovary tissue sections. Ovary tissues were sampled at four key time points across one reproductive year during (A) pre-vitellogenesis (PreVG), (B) early-vitellogenesis (EVG), (C) mid-vitellogenesis (MVG), and (D) post-vitellogenesis (PostVG). Scale bar is 500 microns.

The most advanced clutch of oocytes within white perch ovary tissues sampled in August, November, February, and May were of the pre-vitellogenic stage (PreVG) (Fig 1A), early-vitellogenic stage (EVG) (Fig 1B), mid-vitellogenic stage (MVG) (Fig 1C), and post-vitellogenic stage (PostVG) (Fig 1D), respectively. Total length and weight statistics are provided in Table 1. All values listed represent average ± standard deviation (SD).

**Table 1:** Sexually mature female white perch sampling statistics.

Sampling Month	Oocyte Stage	Weight (g) ± SD	Length (mm) ± SD
August	PreVG	114 ± 59	188 ± 27
November	EVG	183 ± 42	216 ± 16
February	MVG	201 ± 59	227 ± 10
May	PostVG	182 ± 15	217 ± 7

Sexually mature female white perch sampling month, oocyte stage, wet weight, and total length statistics. Values shown are average ± standard deviation (SD).

### Absolute Quantification of Vitellogenins by Protein Cleavage-Isotope Dilution Mass Spectrometry Selected Reaction Monitoring LC-MS/MS

**Fig 2:**
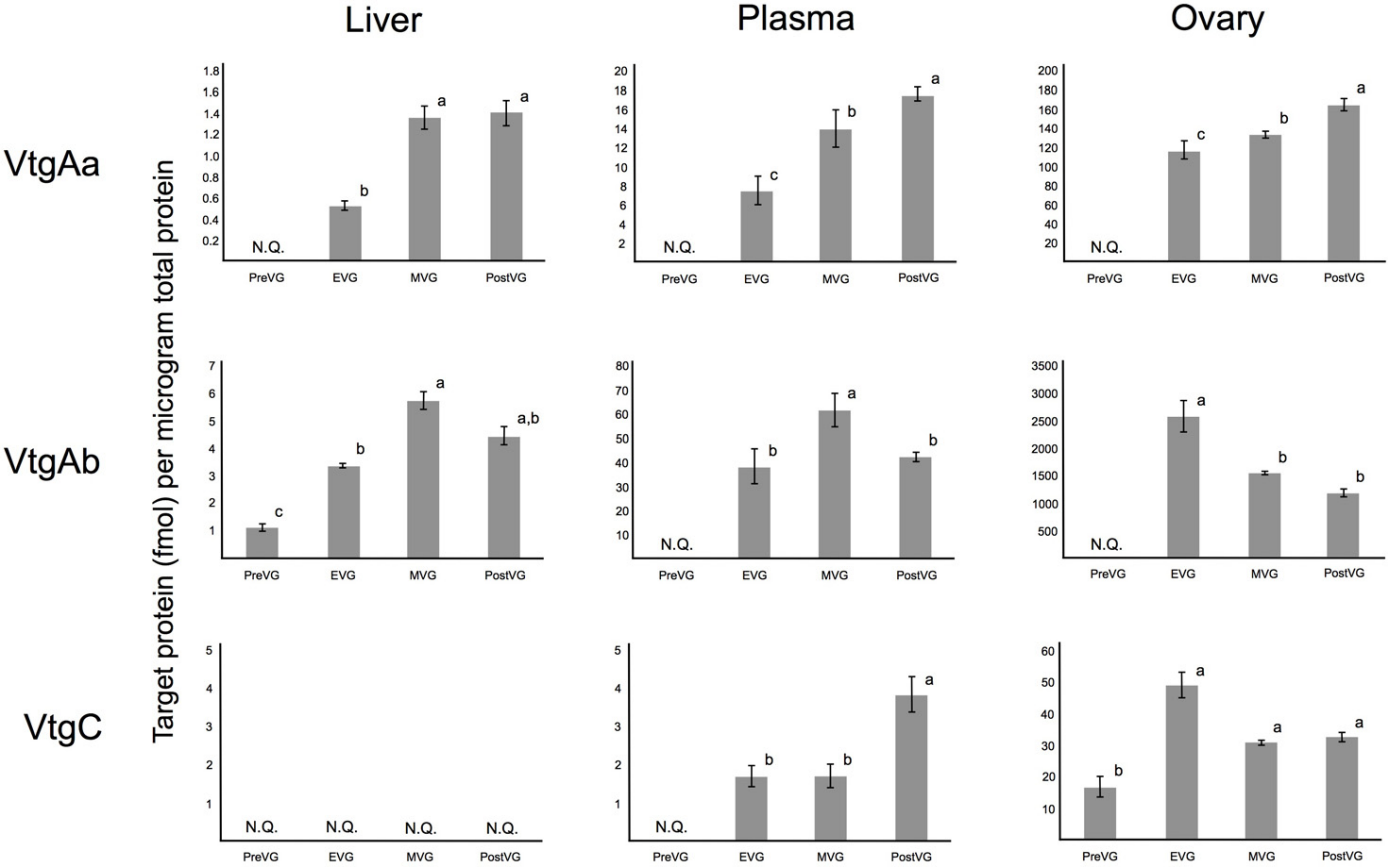
Protein cleavage-isotope dilution mass spectrometry (PC-IDMS) tandem mass spectrometry results for the three white perch vitellogenins (VtgAa, VtgAb, and VtgC). Results from protein cleavage-isotope dilution mass spectrometry (PC-IDMS) tandem mass spectrometry for the three white perch vitellogenins (VtgAa, VtgAb, and VtgC) in female liver, plasma, and ovary tissues sampled across one reproductive year during pre-vitellogenesis (PreVG), early-vitellogenesis (EVG), mid-vitellogenesis (MVG), and post-vitellogenesis (PostVG) across 3 biological replicates. The mean ± SD is shown. “N.Q.” indicates that the native peptide was not quantifiable. Levels not connected by the same letter are significantly different at α = 0.05.

In white perch, VtgAb was the predominant vitellogenin detected in liver, plasma, and ovary during vitellogenesis (Fig 2 and S1 Fig; S1 Table). VtgAb was most abundant during MVG in the liver, plasma, and ovary as measured by PC-IDMS (5.706 ± 0.330, 61.757 ± 3.209, and 1,557.167 ± 27.513 fmol/ug total protein, respectively).

As measured by PC-IDMS, VtgAa was not quantifiable in any PreVG tissue and was most abundant in PostVG liver, plasma, and ovary tissues (1.419 ± 0.118, 17.529 ± 0.757, and 165.617 ± 6.815 fmol/ug total protein) (Fig 2 and S1 Fig; S1 Table).

Vitellogenin C was not quantifiable by PC-IDMS in liver tissues at any stage of oocyte growth (Fig 2 and S1 Fig; S1 Table). VtgC was most abundant in PostVG plasma as measured by PC-IDMS (3.847 ± 0.475 fmol/ug total protein) (Fig 2 and S1 Fig; S1 Table), while it was most abundant in EVG ovary tissues (49.212 ± 4.052 fmol/ug total protein) (Fig 2 and S1 Fig; S1 Table).

In PreVG tissues, only VtgAb could be confidently quantified in liver (Fig 2 and S1 Fig) while only VtgC could be confidently quantified in the ovary (Fig 2 and S1 Fig). None of the vitellogenins were quantifiable in PreVG plasma (Fig 2 and S1 Fig).

The proportional abundance of white perch VtgAa, VtgAb, and VtgC within PostVG ovary tissues as measured by PC-IDMS were 5: 36: 1, respectively (S1 Table). S2 Fig presents example extracted ion chromatograms depicting the co-elution of heavy and light VtgAb peptides (A), native and heavy transitions (B, C), and extracted ion chromatograms depicting the co-elution of heavy and light VtgAb and VtgC peptides that were quantifiable in PreVG ovary tissues (D, E).

Plots of the percent relative standard deviation across biological replicates for the quantification of each vitellogenin in each tissue are provided (S3 Fig).

### White Perch Vitellogenin Western Blotting

**Fig 3:**
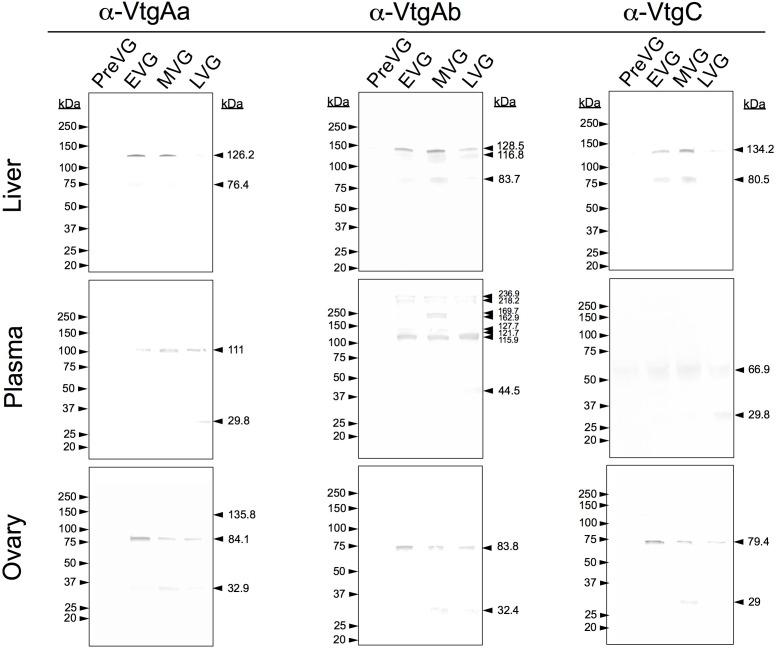
White perch vitellogenin Aa, Ab, and C western blotting. Results of western blotting for the three white perch vitellogenins in female liver, plasma, and ovary tissues sampled across one reproductive year during pre-vitellogenesis (PreVG), early-vitellogenesis (EVG), mid-vitellogenesis (MVG), and post-vitellogenesis (PostVG) pooled from three individuals at each time point.

Tissue samples from three individuals were pooled at each time point in order to perform western blotting. The western blotting results from white perch liver, plasma, and ovary tissues for VtgAa, VtgAb, and VtgC are presented in Fig 3. In the liver, protein bands of 126.2 kDa and 76.4 kDa reacted with α-White Perch VtgAa in EVG and MVG tissues collected in November and February, respectively. Protein bands of 128.5, 116.8, and 83.7 kDa reacted with α-White Perch VtgAb in EVG, MVG, and PostVG liver tissues. Protein bands of 134.2 and 80.5 kDa reacted with α-White Perch VtgC in MVG and EVG liver tissues.

Western blotting of plasma for VtgAa revealed a reactive protein band of 111 kDa during vitellogenesis (November-May). There is also a 29.8 kDa band visible in the PostVG plasma sample. Multiple protein bands were visible in vitellogenic plasma samples stained with α-White Perch VtgAb ranging from 236.9 to 44.5 kDa. There are faint bands of 66.9 and 29.8 kDa in plasma stained with α-White Perch VtgC.

All three vitellogenins were detectable during vitellogenesis in ovary tissues by western blotting. Staining with α-VtgAa produced two bands of 84.1 and 32.9 kDa from early-, mid-, and post-vitellogenic ovary tissues, with a larger 135.8 kDa band present in EVG ovary tissues. Vitellogenin Ab was detected as a band of 83.8 kDa from EVG through PostVG, with a 32.4 kDa band visible in MVG. Similarly, VtgC was detected mainly as one band of 79.4 kDa from EVG through PostVG with a 29 kDa band visible in MVG.

### White Perch Vitellogenin Receptor Western Blotting

**Fig 4:**
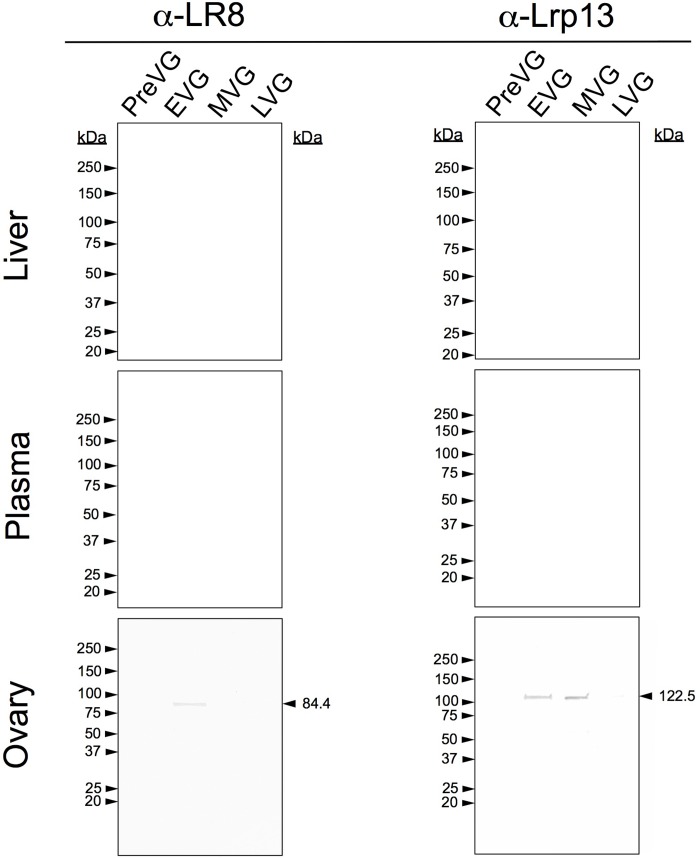
White perch LR8 and Lrp13 western blotting. Results of western blotting for the two white perch vitellogenin receptors in female liver, plasma, and ovary tissues sampled across one reproductive year during pre-vitellogenesis (PreVG), early-vitellogenesis (EVG), mid-vitellogenesis (MVG), and post-vitellogenesis (PostVG) across three biological replicates.

Western blotting for white perch LR8 and Lrp13 indicated that LR8 expression was highest in the ovary during EVG, while Lrp13 expression was highest in the MVG ovary (Fig 4). Neither LR8 nor Lrp13 was detectable in plasma or liver by western blotting (Fig 4).

### White Perch Vitellogenin C Immunohistochemistry

**Fig 5:**
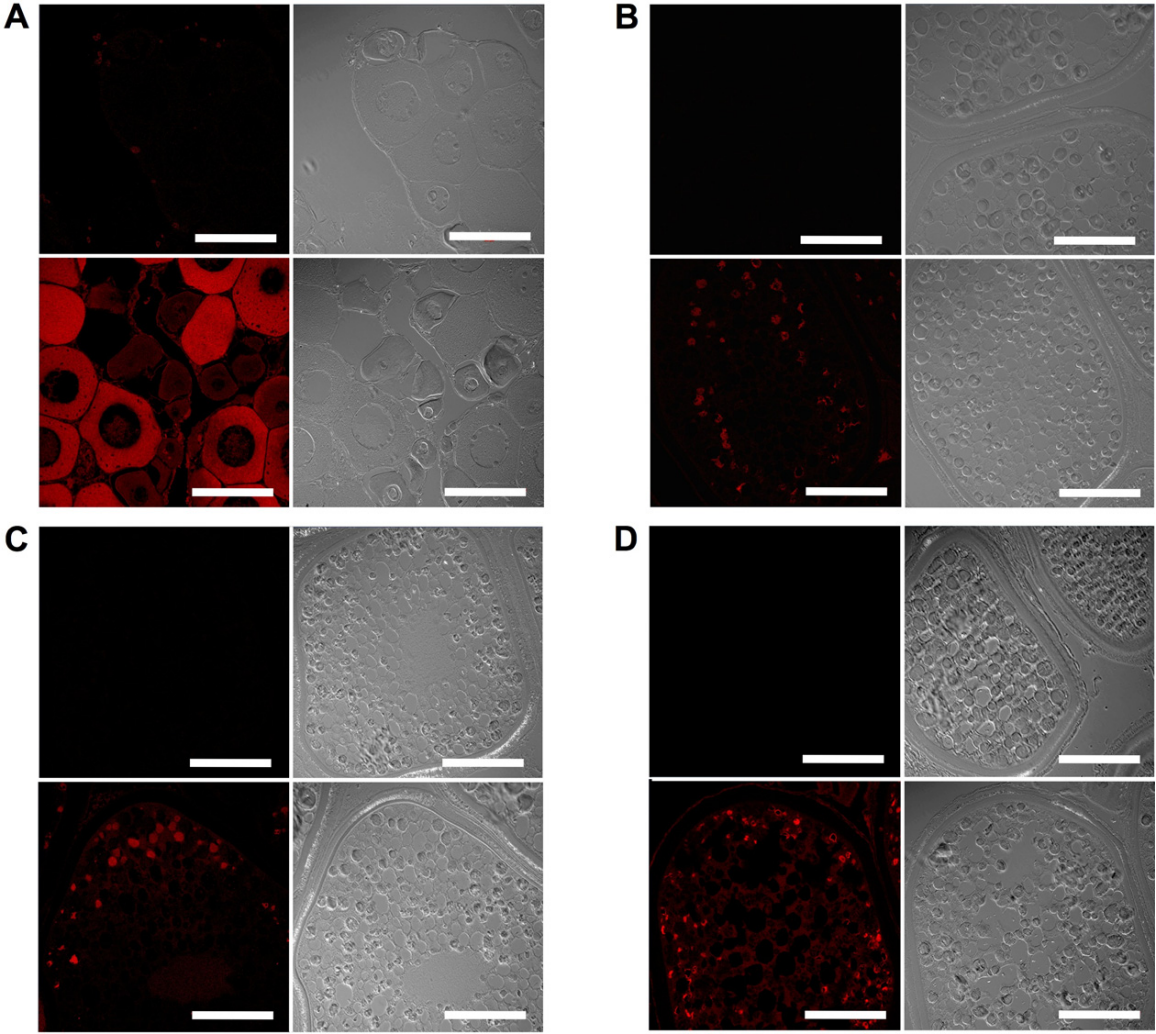
Confocal microscopy of anti-VtgC coupled to DyLight633. Confocal microscopy images of immunohistochemistry of mature female white perch ovary tissues across one reproductive year stained with anti-VtgC coupled to DyLight633: (A) pre-vitellogenic (PreVG), (B) early-vitellogenic (EVG), (C) mid-vitellogenic (MVG), and (D) post-vitellogenic (PostVG) ovary sections.

Confocal microscopy imaging of white perch PreVG ovary sections stained with α-White Perch VtgC covalently coupled to DyLight633 indicates that VtgC is present within primary growth perinucleolar stage oocytes in PreVG ovary tissues (Fig 5A). As oocyte growth progresses into vitellogenesis, VtgC localizes exclusively to lipid droplets (Fig 5B–5D).

### White Perch Vitellogenin C AP-MS/MS

Tandem mass spectrometry confirmed that the VtgC bait protein was highly purified (*data not shown*). Elution from the VtgC-labeled Affi-Gel using buffer containing 10mM suramin yielded a single specific binding protein from the ovary membrane preparation, Y-box binding protein 2a-like (Ybx2a-like). Average peak area for the top three tryptic peptides mapping to Ybx2a-like was 9.75 X 10^6^ for the VtgC-labeled Affi-Gel and was not quantifiable using the control Affi-Gel (Table 2).

**Table 2:** VtgC affinity purification coupled to tandem mass spectrometry.

Elution Buffer	Bait Protein	Striped Bass Ovary Transcriptome ID	Protein ID	Protein MW (kDa)	Sequence Coverage (%)	# Unique Peptides	Peak Area
**Suramin**	**VtgC**	**Contig00578**	**Y-box binding protein 2a-like**	**37.9**	**5.56**	**2**	**9.75E6**
**Suramin**	**Blank**	**Contig00578**	**Y-box binding protein 2a-like**	**37.9**	**0.00**	**0**	**0.00E0**

Results from affinity purification coupled to tandem mass spectrometry using purified white perch VtgC as a bait protein and a white perch ovary membrane preparation as prey.

## Discussion

In this study we present the first absolute quantification by selected reaction monitoring tandem mass spectrometry of white perch vitellogenins in liver, plasma, and ovary sampled at four stages across one reproductive year. Western blotting using antibodies raised against unique, Vtg-specific synthetic peptides largely supports the quantitative mass spectrometry data. In further agreement with the quantitative mass spectrometry data, immunohistochemistry reveals that VtgC is present in PreVG ovary tissues and localizes to lipid droplets in vitellogenic oocytes. Affinity purification coupled to tandem mass spectrometry identified a specific interaction between VtgC and Ybx2a-like protein that was disrupted by suramin elution buffer. Finally, we show that VtgAb is the predominant vitellogenin in the post-vitellogenic perch ovary (86%), with VtgAa and VtgC comprising 12% and ~2% of the Vtg-derived yolk, respectively.

While VtgAb is the predominant perch vitellogenin in liver, plasma, and ovary during vitellogenesis, PC-IDMS also indicates that the proportion of VtgAa accumulated in the PostVG ovary increases from MVG (Fig 2; S1 Fig). A similar pattern of vitellogenin uptake in ovary tissues of striped bass has been reported recently [15], suggesting that the oocytes substantially accumulate VtgAb from EVG through MVG and preferentially accumulate VtgAa from MVG through PostVG. As in the striped bass, accumulation VtgAa and VtgAb by the oocyte is not solely dependent upon the circulating plasma Vtg concentrations. Their accumulation does, however, positively correlate with the expression patterns of LR8 and Lrp13, confirming the likely involvement of these ovary Vtgrs in the uptake of VtgAb and VtgAa, respectively [27].

Neither VtgAa nor VtgAb could be confidently quantified in the PreVG white perch ovary. We were unable to confidently quantify VtgC in white perch liver tissues at any stage of oocyte growth. In our previous study of striped bass vitellogenins, VtgC was also undetectable by nanoLC-MS/MS in liver tissues sampled across one reproductive year, despite comprising over a quarter (26%) of the total vitellogenin accumulated in late-vitellogenic oocytes [15]. Furthermore, the primary site of *vtgc* gene expression was the striped bass liver, indicating that it might be secreted shortly after synthesis and not stored in the tissue, however this has yet to be verified. Although it is present at low levels (17.005 ± 3.209 fmol/ug total protein; S1 Table; S1 Fig), we were able to confidently quantify VtgC in PreVG ovary tissues. Given 1) its limited estrogen responsiveness in white perch and other fish species [38 and 39], 2) its continuous deposition into perch oocytes from PreVG through PostVG (Fig 2 and S1 Fig) [40], 3) its inability to bind the LR8 and Lrp13 vitellogenin receptors in perch (Table 2) [26 and 27] and 4) its species-specific high variation in percent contribution to the mature egg yolk [15], VtgC from Acanthomorph fishes appears to behave more similarly to other lipoproteins than to complete type vitellogenins [41]. It is interesting that striped bass, with 26% VtgC-derived yolk in their mature oocytes, spawn neutrally buoyant eggs, while white perch, with just 2–4% VtgC-derived yolk in their mature oocytes, ovulate adhesive eggs [15]. Further investigation will be required to understand the molecular basis underlying these differences in life history and whether or not they might relate to highly variable amounts of VtgC within mature oocytes of other fish species from diverse lineages.

Western blotting results for white perch VtgAa, VtgAb, and VtgC across all tissues and time points generally confirm the findings of quantitative mass spectrometry (Fig 2 and S1 Fig). Two exceptions, however, were that VtgAb and VtgC were not clearly detected by western blotting in the PreVG liver and ovary, respectively (Fig 3), whereas they were detected by mass spectrometry in these tissues at low levels (Fig 2; S1 Table). Additionally, VtgC was detected in PreVG ovary by immunohistochemistry (see below). Such discrepancies between quantitative mass spectrometry data and western blotting data are not surprising and have been reported for striped bass vitellogenins as well [15]. Given the highly variable performance of antibodies in immunoassays and the high sensitivity of mass spectrometry, the practical utility of validating quantitative mass spectrometry results by western blotting has been brought into question [42].

Our western blotting results are consistent with those in our previous study on striped bass vitellogenins [15, 32]. The antibodies used in the present study were prepared using two unique antigenic synthetic peptides within the LvH domain of each vitellogenin. As such, the α-White Perch Vtg western blots in the present study generally showed fewer reactive bands than those in our previous studies, which utilized antibodies raised against both the LvH and lipovitellin light (LvL) domains of vitellogenins purified from plasma of E_2_-induced mullet (*Mugil cephalus*) in the case of α-Mullet Vtgs [12, 13, 15, 32, 43, 44], and against purified white perch VtgAa, VtgAb, and VtgC in the case of the α-WP Vtgs used by Hiramatsu et al. in 2002 [17]. No reactivity was seen by western blotting in PreVG liver, PreVG plasma, or PreVG ovary samples with any of the antibodies used in this study (Figs 3 and 4).

Western blotting with α-White Perch VtgAa resulted in a major band of 126.2 kDa in the EVG and MVG liver samples, with a faint 76.4 kDa band present in the EVG sample. This banding pattern is consistent with the α-Mullet VtgAa western blotting of striped bass liver samples and the α-WP Vtgs western blotting of striped bass post-vitellogenic and ovulated oocyte extracts in our previous studies [15, 32]. Western blotting of perch plasma showed an apparent VtgAa band of 111 kDa was present in EVG, MVG, and PostVG samples, with a 29.8 kDa band visible in the PostVG sample. Vitellogenins are progressively proteolyzed into smaller yolk proteins during oocyte growth and α-Vtg western blotting, both in the present study and our previous studies, reflects this [15, 32]. The present study confirms that these smaller yolk proteins are derived from the LvH domain. Specifically, α-White Perch VtgAa western blotting of ovary tissues revealed two reactive bands of 84.1 and 32.9 kDa from EVG through PostVG, with an additional band of 135.8 kDa visible in the EVG sample.

Western blotting with α-White Perch VtgAb resulted in bands of 128.5 116.8, and 83.7 kDa in EVG, MVG, and PostVG white perch liver samples. EVG, MVG, and PostVG plasma samples showed reactive bands of 236.9, 218.2, 127.7, 121.7, and 115.9 kDa when stained with α-White Perch VtgAb. The MVG plasma sample had additional reactive bands of 169.7 and 162.9 kDa, while the PostVG sample had an additional faint band of 44.5 kDa. In ovary tissues, α-White Perch VtgAb reacted with an 83.8 kDa band from EVG through PostVG, with a faint 32.4 kDa appearing in the MVG and PostVG samples.

Western blotting with α-White Perch VtgC revealed a band of 134.2 kDa in EVG, MVG, and PostVG white perch liver samples. An additional 80.5 kDa band was faintly visible in the EVG and MVG samples. A diffuse band of 66.9 kDa was faintly visible in PreVG, EVG, MVG, and PostVG plasma samples stained with α-White Perch VtgC. There was an additional faint 29.8 kDa band in the PostVG plasma sample. A 79.4 kDa band was found in EVG, MVG, and PostVG ovary samples when stained with α-White Perch VtgC with an additional 29 kDa band faintly visible in the MVG ovary sample.

Immunohistochemisty (IHC) of PreVG perch ovary tissues using α-White Perch VtgC covalently linked to DyLight633 indicates that VtgC is present in the ooplasm of secondary growth oocytes within the PreVG ovary (Fig 5A). As ovarian maturation progresses into vitellogenesis, IHC reveals that VtgC is primarily sequestered into a ring of lipid droplets in the peripheral ooplasm (Fig 5B–5D). To our knowledge, this represents the first report of VtgC localization within growing oocytes and the first report of a Vtg localizing to a cellular structure other than a yolk inclusion. Presence of VtgC in the lipid droplets suggests an uptake mechanism different from that of VtgAa and VtgAb, which is mediated by the Lrp13 and LR8 ovary receptors.

Using the striped bass ovary transcriptome as a reference database, a single protein, Ybx2a-like, was found to interact specifically with VtgC by AP-MS/MS. This protein specifically eluted with the known disruptor of lipoprotein-lipoprotein receptor interactions, suramin [45]. Y-box-binding proteins are highly expressed in germ cells and bind Y-box elements in the promoters of certain genes and also bind to mRNA transcribed from these genes [46]. A ClustalW dendrogram of vertebrate Y-box-binding proteins (Ybx1, Ybx2, Ybx3, and Ybx2a) places Ybx2a proteins in a distinct group of largely uncharacterized predicted proteins in a number of fish species (S4 Fig). While Ybx1, Ybx2, and Ybx3 are nuclear proteins, *Xenopus* Ybx2a is only present in testis and immature oocytes where it localizes to cytoplasm and has been shown to affect transcription as well as the accumulation of transcripts that it stimulates [47]. Interestingly, Ybx2a-like was detected primarily in the membrane fraction in our previous compartment proteomics study of the white perch ovary [16]. Sequence analysis of the eleven Ybx2a protein sequences presented in S4 Fig indicates that they all lack a transmembrane domain and are likely soluble proteins. While further investigation into the sub-cellular localization, function, and interactions of this protein are needed, it is apparent that Ybx2a-like protein binds VtgC and may act in a receptor or escort function. The absence of a transmembrane domain paired with its presence in the ovary membrane fraction suggest that Ybx2a-like might interact with proteins in the transmembrane system.

In the goldsinny wrasse (*Ctenolabrus rupestris*), a fish species that spawns hyper-pelagic eggs, the VtgAa comprises nearly all of the vitellogenin derived egg yolk [14]. The proportional ratios of accumulated VtgAa: VtgAb, on the other hand, appear to fall within a range of 1: 1 to 1: 7 for striped bass and white perch, respectively. Therefore, differences in VtgAa and VtgAb ratios appear to correlate to differences in egg buoyancy between these two closely related species, as the striped bass spawns neutrally buoyant eggs and the white perch spawns demersal adhesive eggs. In barfin flounder (*Verasper moseri*), a marine species that spawns pelagic eggs, the ratio of VtgAa: VtgAb is 9: 15 [48], however the substantially higher proportion of VtgAb in the egg yolk of white perch may relate to its characteristic demersal eggs. More information regarding the egg yolk compositions of various fishes will be required to fully understand the influence of vitellogenin on egg buoyancy, however it appears that a greater proportion of VtgAb-derived egg yolk is related to the reproductive strategy of spawning demersal eggs.

**Fig 6:**
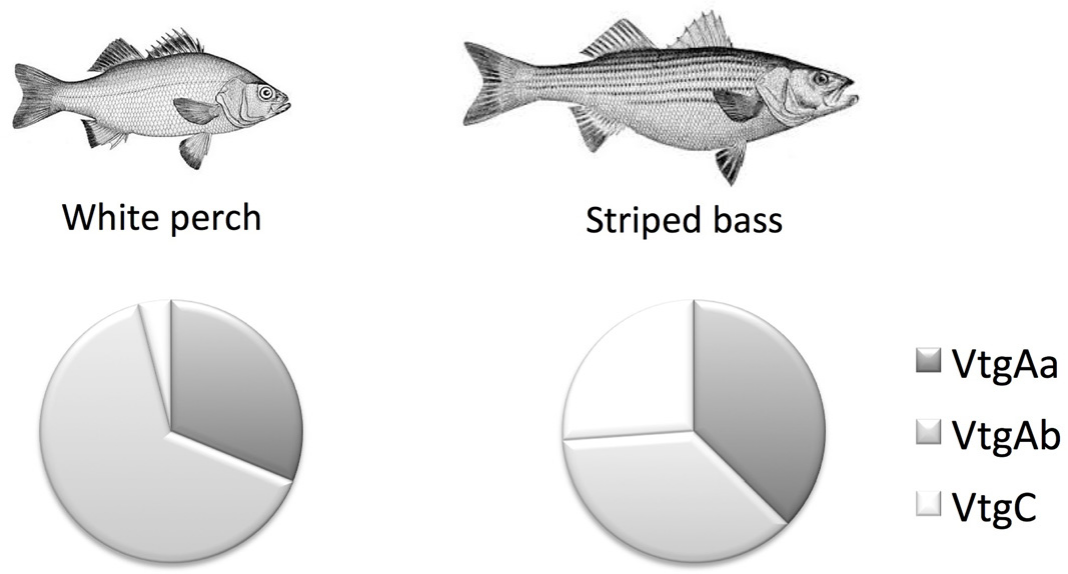
Vitellogenin composition in white perch and striped bass egg yolk. In white perch (*M*. *americana*), yolk proteins derived from VtgC are minor components of the total egg yolk (< 5%), whereas in striped bass (*M*. *saxatilis*) they are major components of the egg yolk (~ 25%). [Williams et al., 2014 (J Exp Zool Part A); Schilling et al., 2014 (J Proteome Res)].

The three Perciform Vtgs are known to undergo different degrees of proteolysis within growing oocytes [8]. While VtgAa and, to a lesser extent, VtgAb are proteolyzed into free amino acids or small peptides, VtgC appears to remain largely intact through oocyte maturation and ovulation and remains available to developing embryos as a nutrient source before they are able to feed on their own. In mosqitofish, for example, the VtgC-derived yolk proteins are the last components of the egg yolk that remain to be consumed by yolk-sac fry [8]. Considering the data that are currently available, it appears that VtgC is the most variable form of vitellogenin within the post-vitellogenic oocytes of Acanthomorph fishes, ranging from ~2% in perch to 26% in striped bass (Fig 6) [15, 17]. Therefore, VtgC composition may relate to other aspects of early life history in these fishes. Further research is required to confirm the function of Ybx2a-like protein or other possible VtgC carriers and to provide the mechanism(s) of VtgC entry into the oocyte and the regulation thereof.

White perch reared indoors in recirculating aquaculture systems (< 5 ppt salinity) had proportional Vtg-derived yolk ratios of 8: 16: 1 in PostVG ovary tissues as reported in our previous study [16]. In the present study, white perch were reared outdoors in a flow through system supplied with estuary creek water (7–15 ppt salinity) and the proportional vitellogenin ratios in PostVG ovary tissues were 5: 36: 1 (S1 Table). The observed proportional ratio differences may be due to differences in culture conditions, differences in salinity, and/or differences in detection methods (i.e. semiquantitative tandem mass spectrometry versus absolute quantification by PC-IDMS selected reaction monitoring tandem mass spectrometry). These differences in the proportional accumulation of Vtgs suggest that there is considerable plasticity in the Vtg-Vtgr system in white perch ovary and that egg yolk formation may be precisely regulated based on the rates of hepatic secretion and receptor-mediated uptake into the ooplasm. Such plasticity may allow fine-tuning of egg buoyancy based upon the specific gravity of the water into which the eggs are to be spawned, which can vary considerably year-to-year depending upon wind, freshwater flow, and the location of the salt front in estuaries [49]. While the Vtgs were not analyzed, eggs in the striped bass varied significantly in size, density, oil globule diameter, and the size of the oil globule relative to oocyte diameter depending upon the watershed from which they were collected [50]. These observations may have important implications in our understanding of the adaptive capabilities of fishes to environmental change, in particular water salinity.

**Fig 7:**
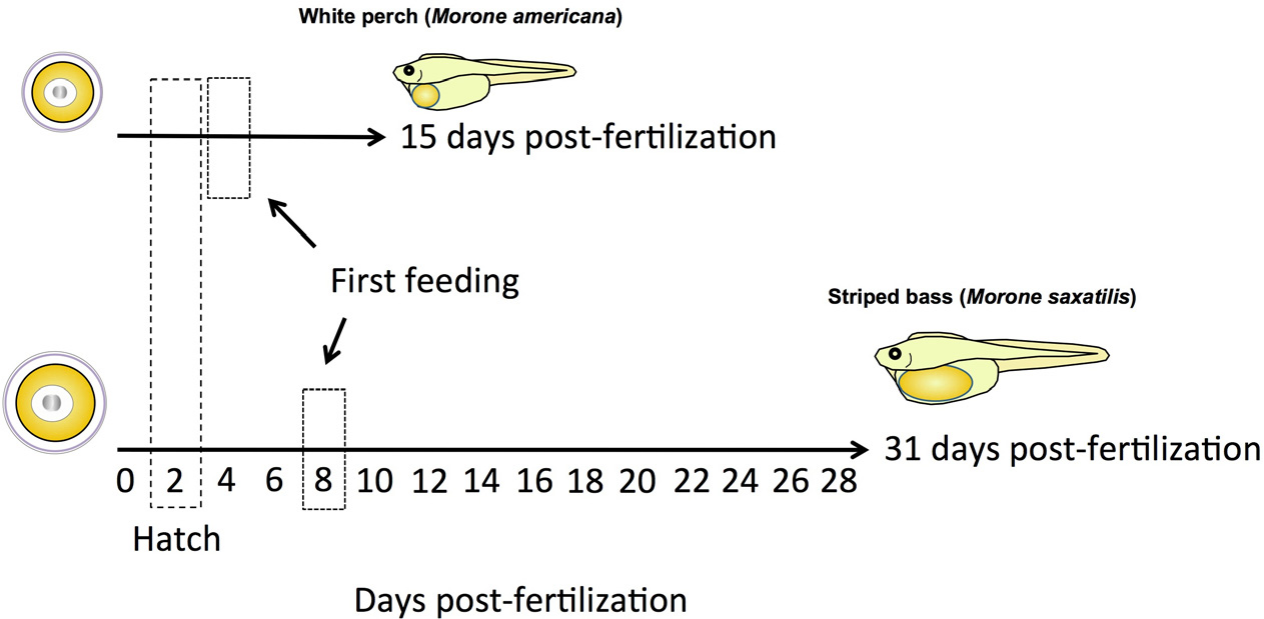
Average survival duration of food-restricted white perch and striped bass larvae. Dashed boxes indicate approximate time of hatching (~2 days) and onset of first feeding (~4 days in white perch, ~8 days in striped bass). [Mansuetti, 1964; Eldridge, et al., 1981; North & Houde, 2003].

White perch and striped bass spawn in similar freshwater or low salinity estuary locations at a similar time of the year and the larvae of both species hatch around 48 hrs post-fertilization [51 and 52]. When food is then restricted or deprived, white perch larvae survive for up to 15 days post-fertilization while striped bass larvae survive for up to 31 days post-fertilization at a similar water temperature (Fig 7) [51 and 52]. Additionally, the time to first feeding of these two species differs, with the white perch and the striped bass larvae beginning to feed at 3–5 days and 7–9 days post-fertilization, respectively. Therefore, although these closely related species share similar time frames during the earliest stages of development (i.e. from fertilization to hatch), the striped bass larvae have an extended developmental window from hatching to first feeding, and these larvae have yolk stores that allow them to survive in the absence of food for twice as long as white perch after hatch. This disparity in early developmental stages post-hatch may relate to differences in VtgC yolk content of the white perch and striped bass eggs, which is ~2% and 26%, respectively. As more data from a wider range of fish species become available, the complexity of the egg yolk system in general and the Vtg-Vtgr system in particular and their relation to the diversity of early life history strategies among fishes may become apparent. Such studies will provide a valuable foundation that will aid in the design of aquaculture husbandry and fisheries management practices, as well as the understanding of gene evolution and environmental adaptation.

## Supporting Information

S1 Fig Absolute quantification by protein cleavage-isotope dilution mass spectrometry (PC-IDMS) of white perch vitellogenins. White perch vitellogenins in the A) liver, B) plasma, and C) ovary tissues sampled across one reproductive year during pre-vitellogenesis (PreVG), early-vitellogenesis (EVG), mid-vitellogenesis (MVG), and post-vitellogenesis (PostVG) were quantified by PC-IDMS across 3 biological replicates. The mean ± SD is shown. “N.Q.” indicates that the native peptide was not quantifiable. Levels not connected by the same letter are significantly different at α = 0.05.
(TIFF)

S2 Fig PC-IDMS extracted ion chromatograms. Extracted ion chromatograms depicting the co-elution of heavy (blue) and light (red) VtgAb (A) native and (B) heavy transitions in post-vitellogenic ovary tissues, and extracted ion chromatograms depicting the co-elution of heavy (blue) and light (red) (C) VtgAb and (D) VtgC peptides that were quantifiable in pre-vitellogenic ovary tissues.
(TIFF)

S3 Fig Mean percent relative standard deviation of white vitellogenins as a measure of PC-IDMS measurement variability. Percent relative standard deviation across 3 biological replicates for absolute quantification of vitellogenin Aa (A), vitellogenin Ab (B), and vitellogenin C (C) in liver, plasma, and ovary across one reproductive year by protein cleavage—isotope dilution selected reaction monitoring tandem mass spectrometry.
(TIFF)

S4 Fig ClustalW dendrogram showing relationships between Y-box-binding protein family polypeptide sequences. GenBank accession numbers are provided. Numbers above each branch are p-distances.
(TIFF)

S1 Table Protein cleavage-isotope dilution mass spectrometry (PC-IDMS) results for the three white perch vitellogenin proteins (VtgAa, VtgAb, and VtgC) in liver, plasma, and ovary. Average amount (femtomoles per microgram total protein) ± SD as determined by protein cleavage-isotope dilution mass spectrometry (PC-IDMS) of the three white perch vitellogenin proteins (VtgAa, VtgAb, and VtgC) in liver, plasma, and ovary during pre-vitellogenesis (PreVG), early-vitellogenesis (EVG), mid-vitellogenesis (MVG), and post-vitellogenesis (PostVG) across 3 biological replicates.
(TIFF)

S2 Table Stable isotope labeled surrogate peptides for the three white perch vitellogenins (VtgAa, VtgAb, and VtgC). White perch vitellogenin gene names, protein identifications, Uniprot accession numbers, peptide sequences, selected reaction monitoring transitions, and collision energies. _6_C^13^ Heavy arginine (R) and lysine (K) residues are indicated by bold text.
(TIFF)

S3 Table Antigenic peptides for white perch vitellogenin Aa, vitellogenin Ab, vitellogenin C, LR8, and Lrp13 used for antibody production. (TIFF)

S1 File PC-IDMS data for white perch vitellogenin Aa, vitellogenin Ab, and vitellogenin C in liver, plasma, and ovary tissues sampled at four time points across one reproductive year and measured across 3 biological replicates at each time point. (XLS)

## References

[1] LaFleur GJ, Byrne BM, Kanungo J, Nelson LD, Greenberg RM, & Wallace RA. Fundulus heteroclitus vitellogenin: the deduced primary structure of a piscine precursor to noncrystalline, liquid-phase yolk protein. J Mol Evol. 1995;41(4): 505–521. 7563139 10.1007/BF00160323

[2] LaFleur GJ, Raldúa D, Fabra M, Carnevali O, Denslow N, Wallace RA, et al Derivation of major yolk proteins from parental vitellogenins and alternative processing during oocyte maturation in *Fundulus heteroclitus* . Biol Reprod. 2005;73(4): 815–824. 10.1095/biolreprod.105.041335 15930322

[3] Matsubara T, Ohkubo N, Andoh T, Sullivan CV, Hara A. Two forms of vitellogenin, yielding two distinct lipovitellins, play different roles during oocyte maturation and early development of barfin flounder, *Verasper moseri*, a marine teleost that spawns pelagic eggs. Dev Biol. 1999;213(1): 18–32. 10.1006/dbio.1999.9365 10452844

[4] Reith M, Munholland J, Kelly J, Finn RN, Fyhn HJR. Lipovitellins derived from two forms of vitellogenin are differentially processed during oocyte maturation in haddock (*Melanogrammus aeglefinus*). J Exp Zool. 2001;291(1): 58–67. 10.1002/jez.5 11335916

[5] Wang H, Yan T, Tan J, Gong Z. A zebrafish vitellogenin gene (vg3) encodes a novel vitellogenin without a phosvitin domain and may represent a primitive vertebrate vitellogenin gene. Gene. 2000;256(1–2): 303–10. 11054560 10.1016/s0378-1119(00)00376-0

[6] Wang H, Tan JTT, Emelyanov A, Korzh V, Gong Z. Hepatic and extrahepatic expression of vitellogenin genes in the zebrafish, Danio rerio. Gene. 2005;356: 91–100. 10.1016/j.gene.2005.03.041 15979250

[7] Sawaguchi S, Koya Y, Yoshizaki N, Ohkubo N, Andoh T, Hiramatsu N, et al Multiple vitellogenins (Vgs) in mosquitofish (*Gambusia affinis*): identification and characterization of three functional Vg genes and their circulating and yolk protein products. Biol Reprod. 2005;72(4): 1045–1060. 10.1095/biolreprod.104.037895 15616220

[8] Sawaguchi S, Ohkubo N, Koya Y, Matsubara T. Incorporation and utilization of multiple forms of vitellogenin and their derivative yolk proteins during vitellogenesis and embryonic development in the mosquitofish, *Gambusia affinis* . Zool Sci. 2005;22(6): 701–710. 15988167 10.2108/zsj.22.701

[9] Sawaguchi S, Kagawa H, Ohkubo N, Hiramatsu N, Sullivan CV, Matsubara T. Molecular characterization of three forms of vitellogenin and their yolk protein products during oocyte growth and maturation in red seabream (*Pagrus major*), a marine teleost spawning pelagic eggs. Mol Reprod Dev. 2006;73(6): 719–736. 10.1002/mrd.20446 16541459

[10] Finn RN. The maturational disassembly and differential proteolysis of paralogous vitellogenins in a marine pelagophil teleost: a conserved mechanism of oocyte hydration. Biol. Reprod. 2007;76(6): 936–948. 10.1095/biolreprod.106.055772 17314318

[11] Finn RN, Kristoffersen BA. Vertebrate Vitellogenin Gene Duplication in Relation to the “3R Hypothesis”: Correlation to the Pelagic Egg and the Oceanic Radiation of Teleosts. PLoS ONE. 2007;2(1): e169 10.1371/journal.pone.0000169 17245445 PMC1770952

[12] Amano H, Fujita T, Hiramatsu N, Sawaguchi S, Matsubara T, Sullivan CV, et al Purification of multiple vitellogenins in grey mullet (*Mugil cephalus*). Mar Biol. 2007;152(6): 1215–1225. 10.1007/s00227-007-0768-z

[13] Amano H, Fujita T, Hiramatsu N, Kagawa H, Matsubara T, Sullivan CV, et al Multiple vitellogenin-derived yolk proteins in gray mullet (*Mugil cephalus*): disparate proteolytic patterns associated with ovarian follicle maturation. Mol Reprod Dev. 2008;75(8): 1307–1317. 10.1002/mrd.20864 18324616

[14] Kolarevic J, Nerland A, Nilsen F, Finn RN. Goldsinny wrasse (*Ctenolabrus rupestris*) is an extreme vtgAa-type pelagophil teleost. Mol Reprod Dev. 2008;75(6): 1011–1020. 10.1002/mrd.20845 18163443

[15] Williams VN, Reading BJ, Amano H, Hiramatsu N, Schilling J, Salger SA, et al Proportional accumulation of yolk proteins derived from multiple vitellogenins is precisely regulated during vitellogenesis in striped bass (*Morone saxatilis*). J Exp Zool. 2014;321(6): 301–315. 10.1002/jez.1859 24648375

[16] Schilling J, Nepomuceno A, Schaff JE, Muddiman DC, Daniels HV, Reading BJ. Compartment proteomics analysis of white perch (*Morone americana*) ovary using support vector machines. J Proteome Res. 2014;13: 1515–1526. 10.1021/pr401067g 24494930

[17] Hiramatsu N, Matsubara T, Hara A, Donato DM, Hiramatsu K, Denslow ND, et al Identification, purification and classification of multiple forms of vitellogenin from white perch (*Morone americana*). Fish Physiol Biochem. 2002;26(4): 355–370. 10.1023/B:FISH.0000009266.58556.9a

[18] Garcia J, Munro ES, Monte MM, Fourrier MCS, Whitelaw J, Smail DA, et al Atlantic salmon (*Salmo salar* L.) serum vitellogenin neutralises infectivity of infectious pancreatic necrosis virus (IPNV). Fish Shellfish Immun. 2010;29(2): 293–297. 10.1016/j.fsi.2010.04.010 20420921

[19] Zhang S, Wang S, Li H, Li L. Vitellogenin, a multivalent sensor and an antimicrobial effector. Int J Biochem Cell B. 2011;43(3): 303–305. 10.1016/j.biocel.2010.11.003 21075213

[20] Wang S, Wang Y, Ma J, Ding Y, Zhang S. Phosvitin Plays a Critical Role in the Immunity of Zebrafish Embryos via Acting as a Pattern Recognition Receptor and an Antimicrobial Effector. J Biol Chem. 2011;286(25): 22653–22664. 10.1074/jbc.M111.247635 21531722 PMC3121409

[21] Zhang J and Zhang S. Lipovitellin is a Non-Self Recognition Receptor with Opsonic Activity. Mar Biotechnol. 2011;13:441–450. 10.1007/s10126-010-9315-x 20857311

[22] Li Z, Zhang S, Liu Q. Vitellogenin Functions as a Multivalent Pattern Recognition Receptor with an Opsonic Activity. PLOS ONE. 2008;3(4): e1940 10.1371/journal.pone.0001940 18398466 PMC2277463

[23] Reading BJ, Hiramatsu N, Sawaguchi S, Matsubara T, Hara A, Lively MO, et al Conserved and variant molecular and functional features of multiple egg yolk precursor proteins (vitellogenins) in white perch (*Morone americana*) and other teleosts. Mar Biotechnol. 2009;11(2): 169–187. 10.1007/s10126-008-9133-6 18766402

[24] Finn RN, Kolarevic J, Kongshaug H, Nilsen F. Evolution and differential expression of a vertebrate vitellogenin gene cluster. BMC Evol Biol. 2009;9: 2 10.1186/1471-2148-9-2 19123940 PMC2632621

[25] Hiramatsu N, Luo W, Reading BJ, Sullivan CV, Mizuta H, Ryu YW, et al Multiple ovarian lipoprotein receptors in teleosts. Fish Physiol Biochem. 2013;39: 29–32. 10.1007/s10695-012-9612-6 22327553

[26] Reading BJ, Hiramatsu N, Sullivan CV. Disparate binding of three types of vitellogenin to multiple forms of vitellogenin receptor in white perch. Biol Reprod. 2011;84(2): 392–399. 10.1095/biolreprod.110.087981 20926805

[27] Reading BJ, Hiramatsu N, Schilling J, Molloy KT, Glassbrook N, Mizuta H, et al Lrp13 is a novel vertebrate lipoprotein receptor that binds vitellogenins in teleost fishes. J Lipid Res. 2014;55(11): 2287–2295. 10.1194/jlr.M050286 25217480 PMC4617131

[28] Andrews Kingon GL, Petitte JN, Muddiman DC, Hawkridge AM. Multi-peptide nLC-PC-IDMS-SRM-based assay for the quantification of biomarkers in the chicken ovarian cancer model. Methods. 2013;61(3): 323–330. 10.1016/j.ymeth.2013.04.004 23603217 PMC4163925

[29] Cohen AM, Jahouh F, Sioud S. Quantification of Greenland halibut serum vitellogenin: a trip from the deep sea to the mass spectrometer. Rapid Commun Mass Sp. 2009;23: 1049–1060. 10.1002/rcm.3966 19263406

[30] Cohen AM, Mansour AA, Banoub JH. Absolute quantification of Atlantic salmon and rainbow trout vitellogenin by the “signature peptide” approach using electrospray ionization QqToF tandem mass spectrometry. J Mass Spectrom. 2006;41(5): 646–658. 10.1002/jms.1023 16541402

[31] Plumel MI, Wasselin T, Plot V, Strub JM, Van Dorsselaer A, Carapito C, et al Mass spectrometry-based sequencing and SRM-based quantitation of two novel vitellogenin isoforms in the leatherback sea turtle (*Dermochelys coriacea*). J Proteome Res. 2013;12(9): 4122–4135. 10.1021/pr400444m 23837631

[32] Williams VN, Reading BJ, Hiramatsu N, Amano H, Glassbrook N, Hara A, et al Multiple vitellogenins and product yolk proteins in striped bass, *Morone saxatilis*: molecular characterization and processing during oocyte growth and maturation. Fish Physiol Biochem. 2013;40(2): 395–415. 10.1007/s10695-013-9852-0 24005815

[33] Zhang F, Bartels MJ, Brodeur JC, Woodburn KB. Quantitative measurement of fathead minnow vitellogenin by liquid chromatography combined with tandem mass spectrometry using a signature peptide of vitellogenin. Environ Toxicol Chem. 2004;23(6): 1408–1415. 10.1897/03-425 15376526

[34] Jackson LF, Sullivan CV. Reproduction of white perch: the annual gametogenic cycle. T Am Fish Soc. 1995;124(4): 563–577. 10.1577/1548-8659(1995)124<0563:ROWPTA>2.3.CO;2

[35] Bradford MM. A rapid and sensitive method for the quantitation of microgram quantities of protein utilizing the principle of protein-dye binding. Anal Biochem. 1976;72: 248–254. 942051 10.1016/0003-2697(76)90527-3

[36] Noble JE, Bailey MJ. Quantitation of Protein. Method Enzymol. 2009;463: 73–95. 10.1016/S0076-6879(09)63008-1 19892168

[37] MacLean B, Tomazela DM, Shulman N, Chambers M, Finney GL, Frewen B, et al Skyline: an open source document editor for creating and analyzing targeted proteomics experiments. Bioinformatics. 2010;26(7): 966–968. 10.1093/bioinformatics/btq054 20147306 PMC2844992

[38] Schilling J, Neopmuceno AI, Planchart A, Yoder JA, Kelly RM, Muddiman DC, et al Machine Learning Reveals Sex-Specific 17β-Estradiol-Responsive Expression Patterns in White Perch (*Morone americana*) Plasma Proteins. Proteomics. 2015;15: 2678–2690. 10.1002/pmic.201400606 25900664 PMC5765861

[39] Ohkubo N, Mochida K, Adachi S, Hara A, Hotta K, Nakamura Y, et al Development of enzyme-linked immunosorbent assays for two forms of vitellogenin in Japanese common goby (*Acanthogobius flavimanus*). Gen Comp Endocrinol. 2003; 131(3): 353–364. 12714018 10.1016/s0016-6480(03)00035-2

[40] Reading BJ, Sullivan CV. Chapter 257: Vitellogenesis in Fishes In: Ferrell AP, editor-in-chief. Encyclopedia of Fish Physiology: From Genome to Environment. Maryland Heights: Elsevier; 2011 pp. 635–646.

[41] Babin PJ, Vernier JM. Plasma lipoproteins in fish. J Lipid Res. 1989;30: 467–489. 2666541

[42] Aebersold R, Burlingame AL, Bradshaw RA. Western Blots versus Selected Reaction Monitoring Assays: Time to Turn the Tables? Mol Cell Proteomics. 2013;12(9): 2381–2382. 10.1074/mcp.E113.031658 23756428 PMC3769317

[43] Amano H, Fujita T, Hiramatsu N, Shimizu M, Sawaguchi S, Matsubara T, et al Egg yolk proteins in gray mullet (*Mugil cephalus*): purification and classification of multiple lipovitellins and other vitellogenin-derived yolk proteins and molecular cloning of the parent vitellogenin genes. J Exp Zool A Ecol Genet Physiol. 2007;307(6): 324–341. 10.1002/jez.388 17480036

[44] Amano H, Fujita T, Hiramatsu N, Kagawa H, Matsubara T, Sullivan CV, et al Multiple vitellogenin-derived yolk proteins in gray mullet (*Mugil cephalus*): disparate proteolytic patterns associated with ovarian follicle maturation. Mol Reprod Dev. 2008;75(8): 1307–1317. 10.1002/mrd.20864 18324616

[45] Brown SA, Via DP, Gotto AM, Bradley WA, Gianturco SH. Apolipoprotein E-mediated binding of hypertriglyceridemic very low density lipoproteins to isolated low density lipoprotein receptors detected by ligand blotting. Biochem Biophys Res Comm. 1986;139(1): 333–340. 3094511 10.1016/s0006-291x(86)80118-8

[46] Safran M, Dalah I, Alexander J, Rosen N, Iny Stein T, Shmoish M, et al GeneCards Version 3: the human gene integrator. Database (Oxford). 2010;2010: baq020 10.1093/database/baq020 20689021 PMC2938269

[47] Tafuri SR, Wolffe AP. Xenopus Y-box transcription factors: molecular cloning, functional analysis and developmental regulation. Proc. Natl. Acad. Sci. USA. 1990;87: 9028–9032. 2247479 10.1073/pnas.87.22.9028 PMC55094

[48] Sawaguchi S, Ohkubo N, Amano H, Hiramatsu N, Hara A, Sullivan CV, et al Controlled accumulation of multiple vitellogenins into oocytes during vitellogenesis in the barfin flounder, *Verasper moseri* . Cybium Int J Ichthyol. 2008;32(suppl 2): 262.

[49] North EW, Houde ED. Linking ETM physics zooplankton prey and fish early life histories to striped bass *Morone saxatilis* and white perch *M*. *americana* recruitment. Mar Ecol-Prog Ser. 2003;260: 219–236.

[50] Bergey L, Rulifson RA, Gallagher ML, Overton AS. Variability of Atlantic Coast Striped Bass Egg Characteristics. N Am J Fish Manage. 2003;23(2): 558–572.

[51] Eldridge MB, Whipple JA, Eng D, Bowers MJ, Jarvis BM. Effects of Food and Feeding Factors on Laboratory-Reared Striped Bass Larvae. T Am Fish Soc. 1981;110(1): 111–120. 10.1577/1548-8659(1981)110<111:EOFAFF>2.0.CO;2

[52] Mansueti RJ. Eggs, Larvae, and Young of the White Perch, *Roccus americanus*, with Comments on Its Ecology in the Estuary. Chesapeake Sci. 1964;5(1/2), 3–45. 10.2307/1350789

