# Supplementary figures and images for: Mechanisms of Egg Yolk Formation and Implications on Early Life History of White Perch (Morone americana)
Source: PLoS One. 2015 Nov 18;10(11):e0143225. doi: 10.1371/journal.pone.0143225 (PMC4651544; doi:10.1371/journal.pone.0143225)

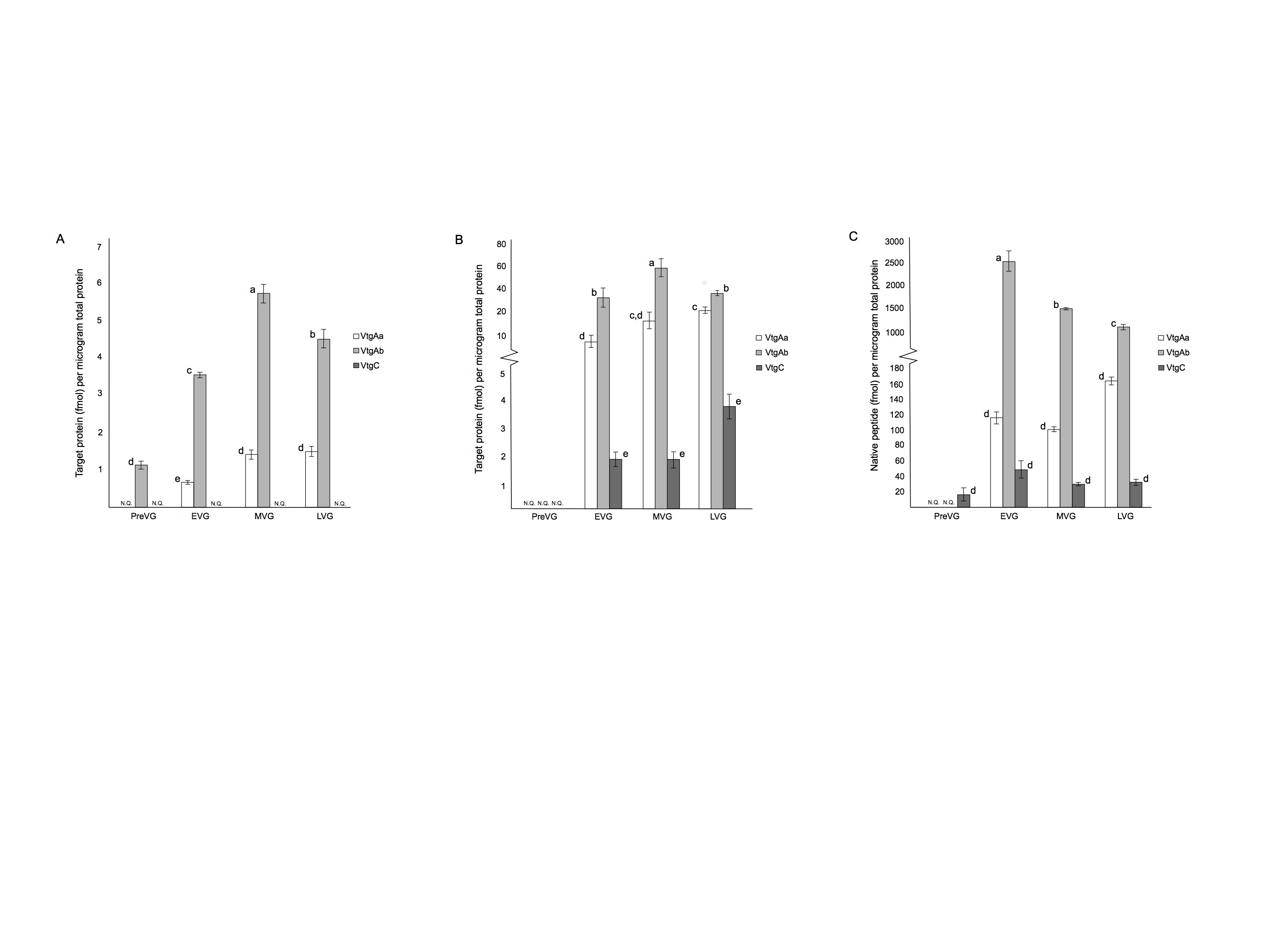

Supplement: S1 Fig — White perch vitellogenins in the A) liver, B) plasma, and C) ovary tissues sampled across one reproductive year during pre-vitellogenesis (PreVG), early-vitellogenesis (EVG), mid-vitellogenesis (MVG), and post-vitellogenesis (PostVG) were quantified by PC-IDMS across 3 biological replicates. The mean ± SD is shown. “N.Q.” indicates that the native peptide was not quantifiable. Levels not connected by the same letter are significantly different at α = 0.05. (TIFF) [file pone.0143225.s001.tiff]

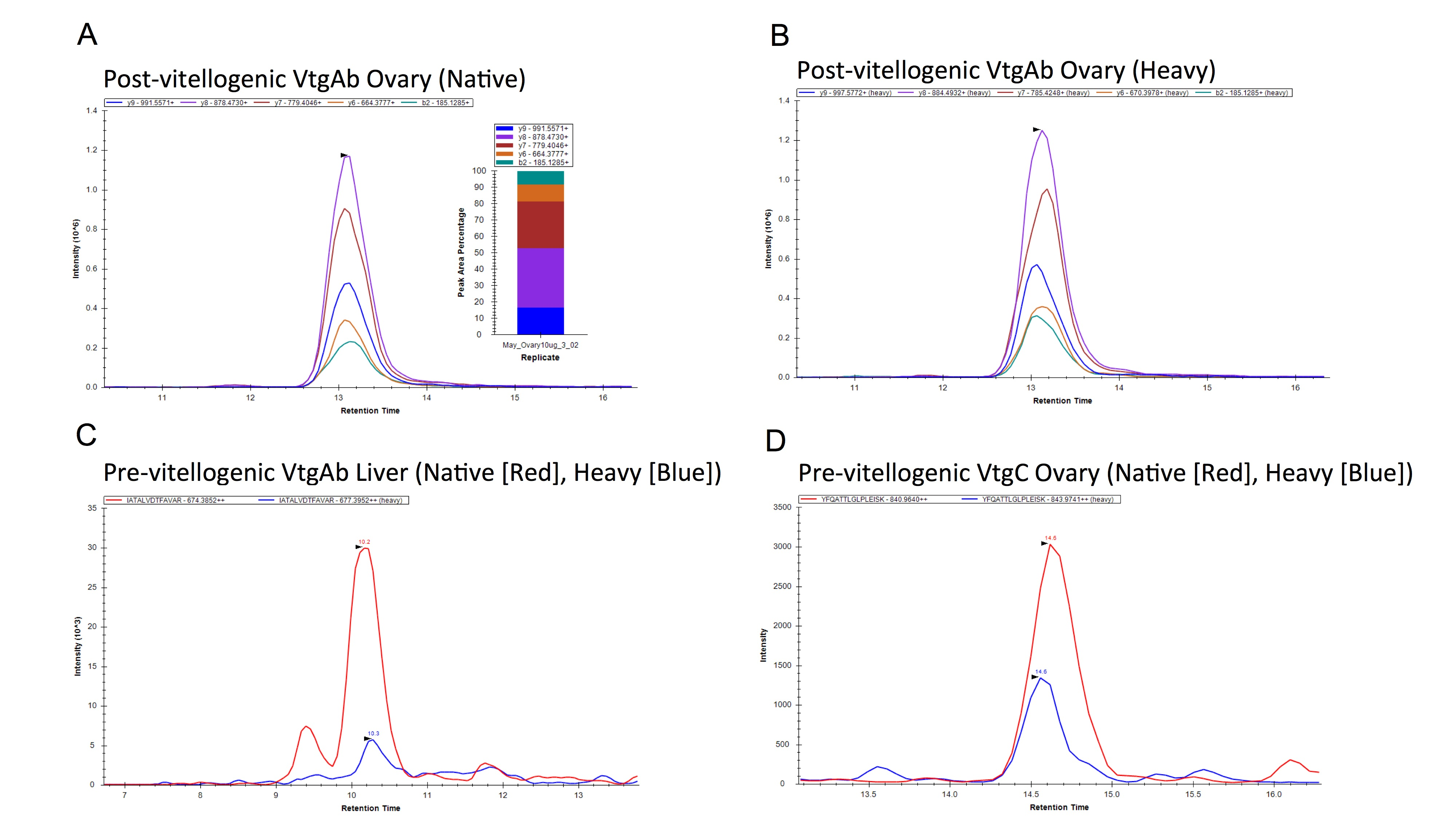

Supplement: S2 Fig — Extracted ion chromatograms depicting the co-elution of heavy (blue) and light (red) VtgAb (A) native and (B) heavy transitions in post-vitellogenic ovary tissues, and extracted ion chromatograms depicting the co-elution of heavy (blue) and light (red) (C) VtgAb and (D) VtgC peptides that were quantifiable in pre-vitellogenic ovary tissues. (TIFF) [file pone.0143225.s002.tiff]

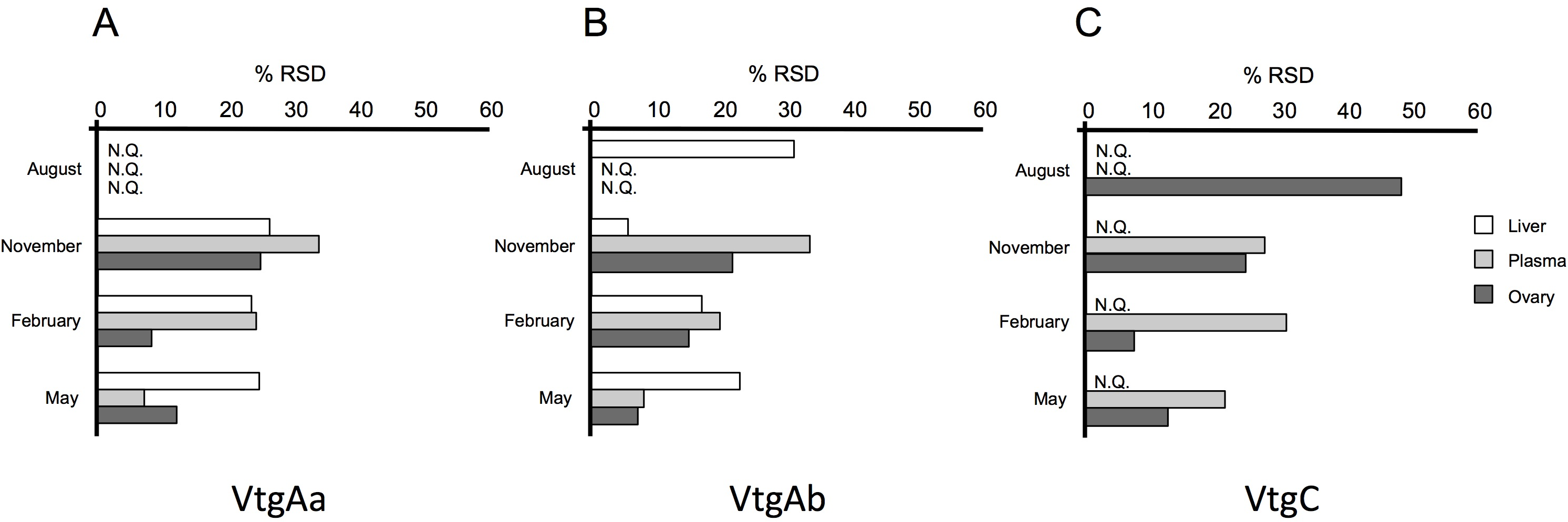

Supplement: S3 Fig — Percent relative standard deviation across 3 biological replicates for absolute quantification of vitellogenin Aa (A), vitellogenin Ab (B), and vitellogenin C (C) in liver, plasma, and ovary across one reproductive year by protein cleavage—isotope dilution selected reaction monitoring tandem mass spectrometry. (TIFF) [file pone.0143225.s003.tiff]

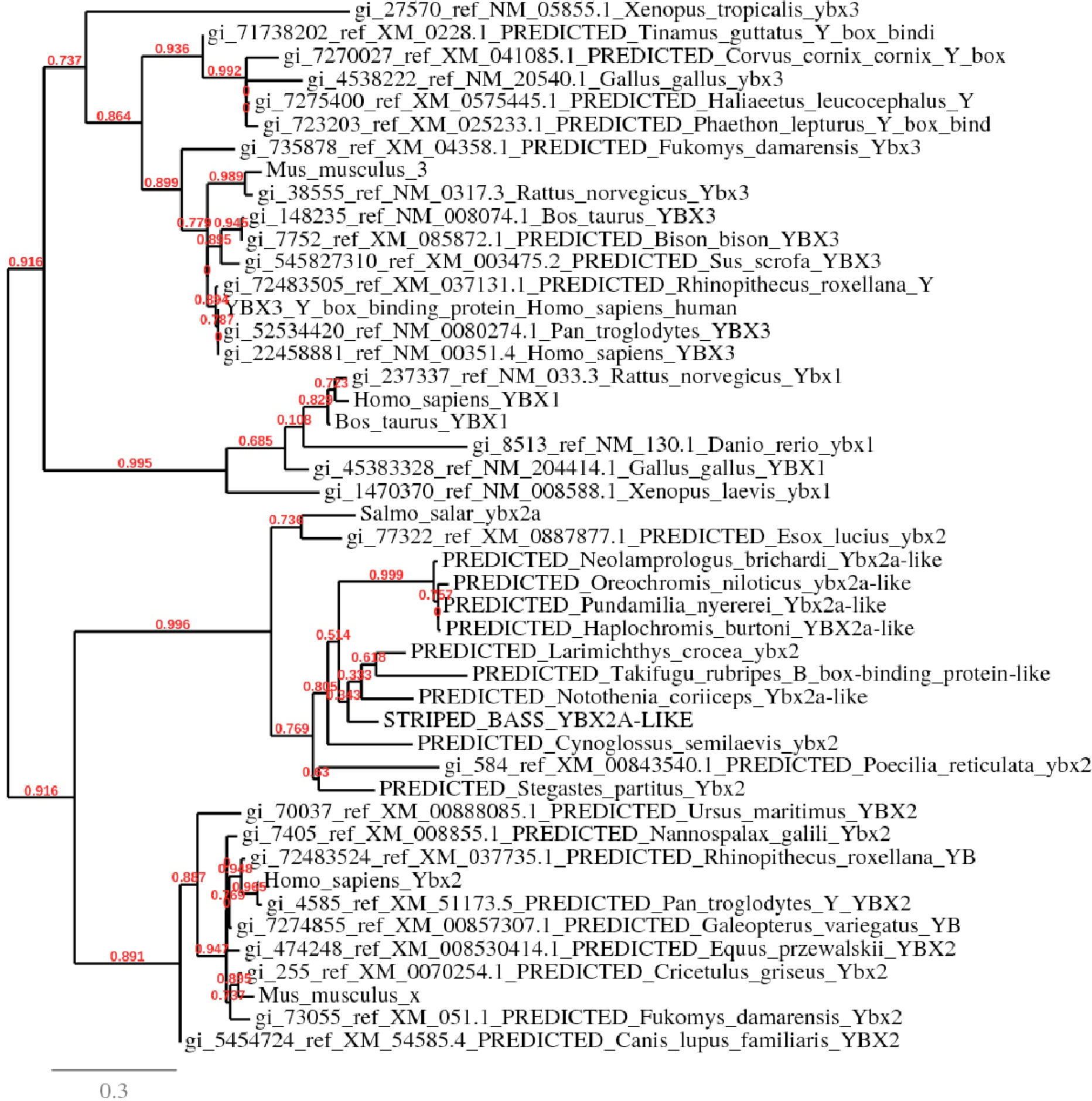

Supplement: S4 Fig — GenBank accession numbers are provided. Numbers above each branch are p-distances. (TIFF) [file pone.0143225.s004.tiff]

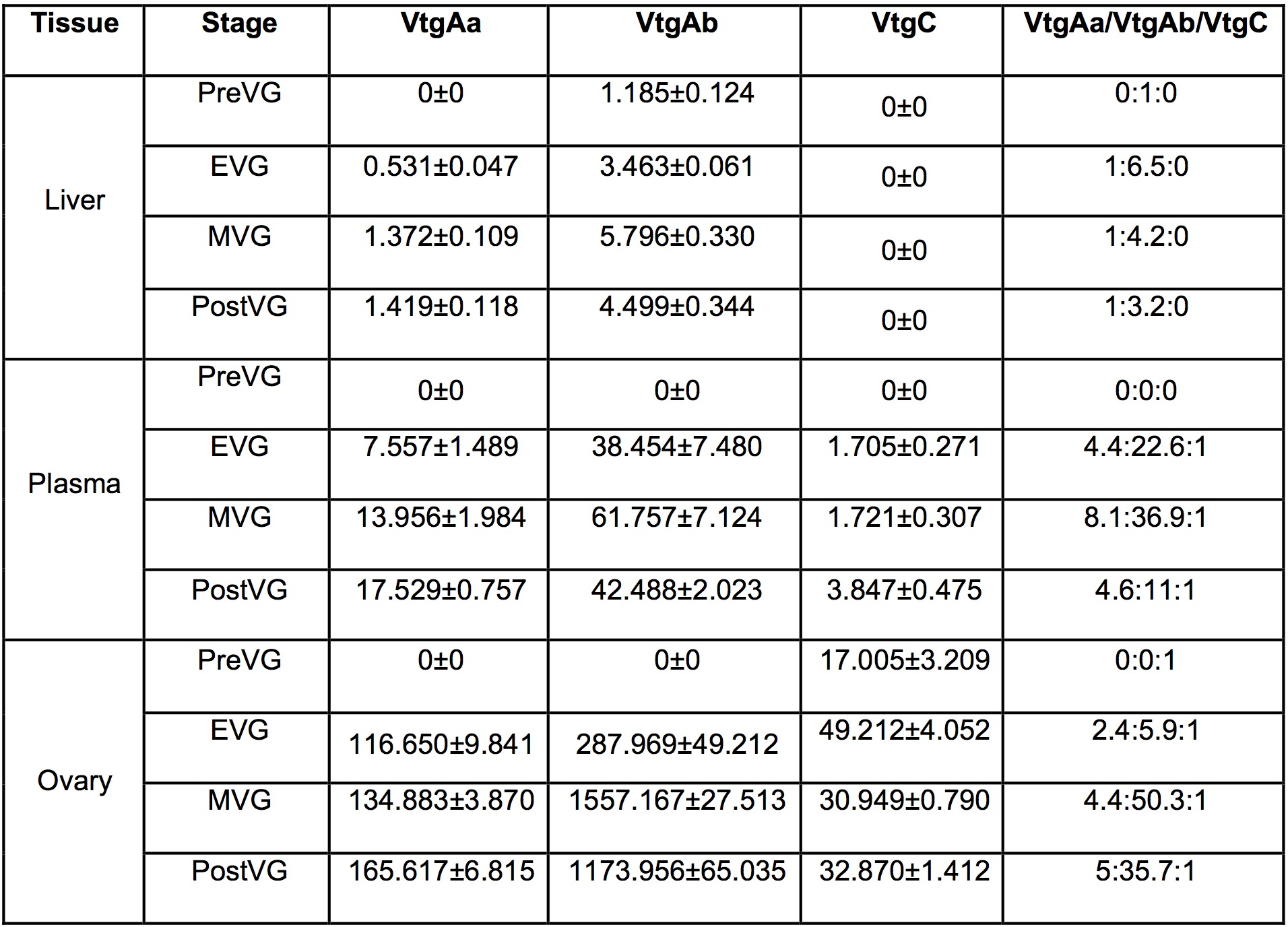

Supplement: S1 Table — Average amount (femtomoles per microgram total protein) ± SD as determined by protein cleavage-isotope dilution mass spectrometry (PC-IDMS) of the three white perch vitellogenin proteins (VtgAa, VtgAb, and VtgC) in liver, plasma, and ovary during pre-vitellogenesis (PreVG), early-vitellogenesis (EVG), mid-vitellogenesis (MVG), and post-vitellogenesis (PostVG) across 3 biological replicates. (TIFF) [file pone.0143225.s005.tiff]

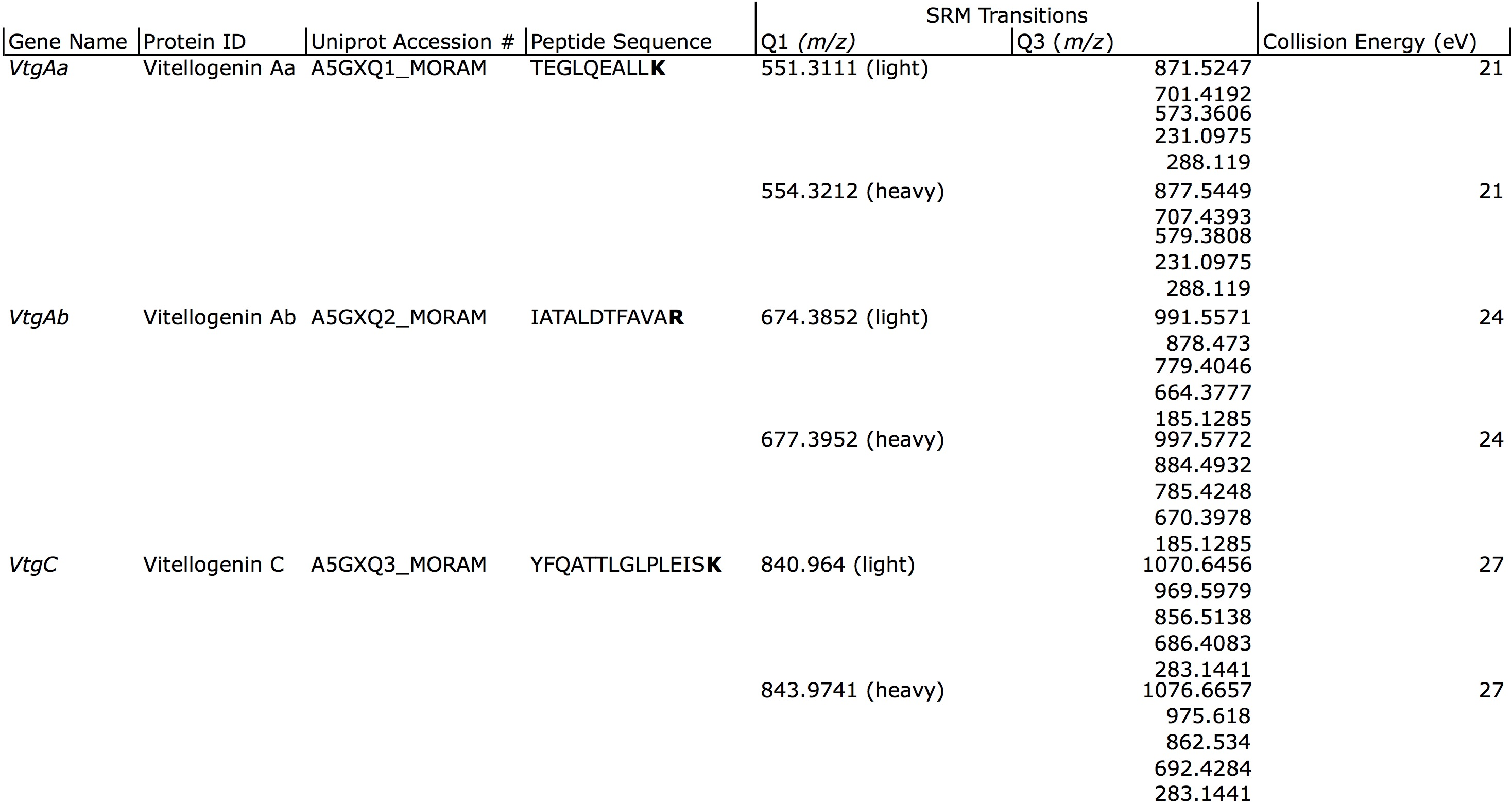

Supplement: S2 Table — White perch vitellogenin gene names, protein identifications, Uniprot accession numbers, peptide sequences, selected reaction monitoring transitions, and collision energies. 6C13 Heavy arginine (R) and lysine (K) residues are indicated by bold text. (TIFF) [file pone.0143225.s006.tiff]

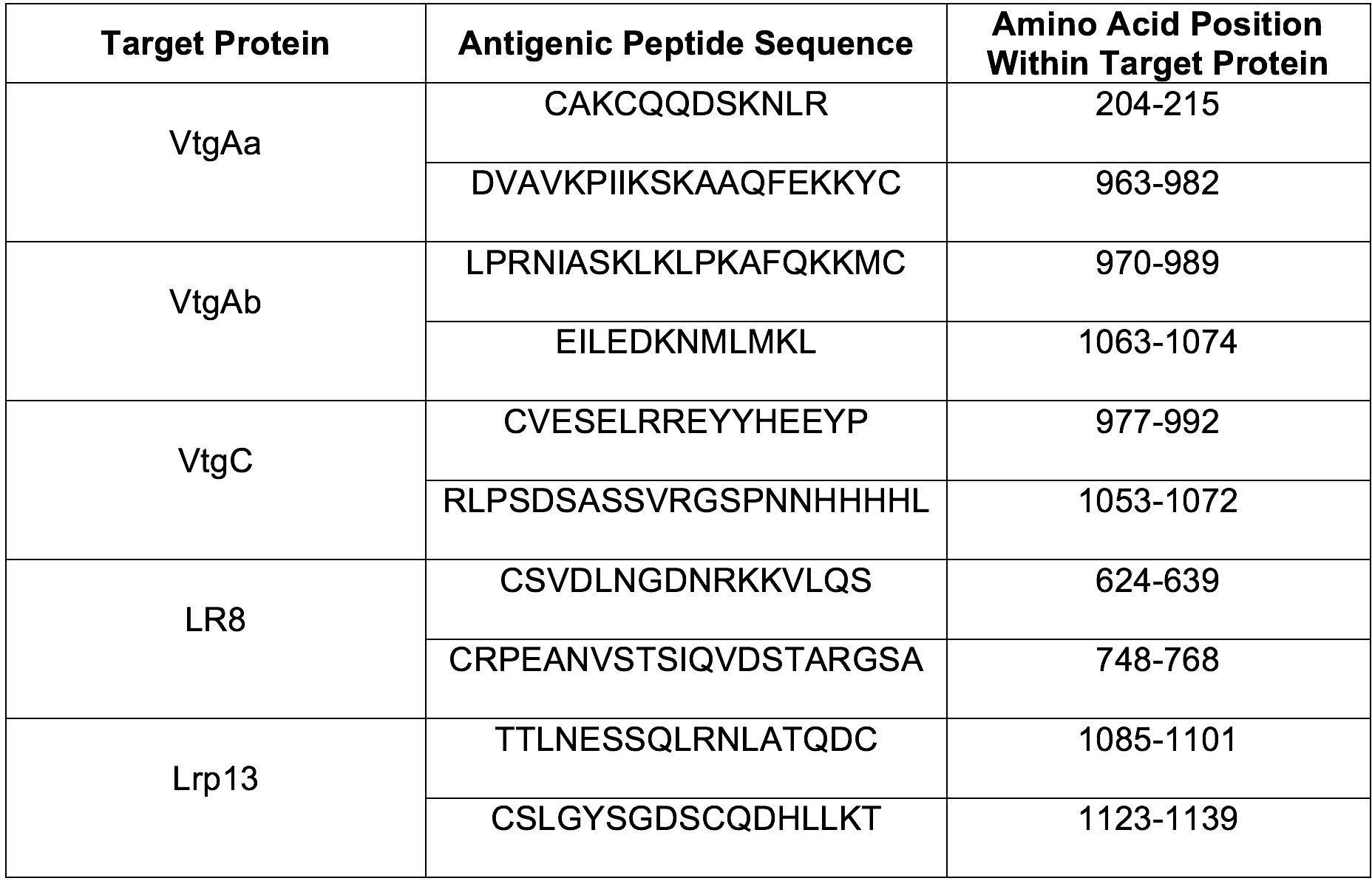

Supplement: S3 Table — (TIFF) [file pone.0143225.s007.tiff]
